# Progress in reducing socioeconomic inequalities in the use of modern contraceptives in 48 focus countries as part of the FP2030 initiative between 1990 and 2020: a population-based analysis

**DOI:** 10.1016/S2214-109X(24)00424-8

**Published:** 2024-12-18

**Authors:** Carolina Cardona, Jean Christophe Rusatira, Carolina Salmeron, Michelle Martinez-Baack, Jose G Rimon, Philip Anglewicz, Saifuddin Ahmed

**Affiliations:** a Department of Population, Family and Reproductive Health, Bloomberg School of Public Health, Johns Hopkins University, Baltimore, MD, USA; b The Jacob and Terese Hershey Foundation, Houston, TX, USA

## Abstract

**Background:**

Despite increases in modern contraception use, socioeconomic inequalities in family planning persist. In this study, we aimed to measure progress in reducing socioeconomic inequalities in modern contraceptive prevalence rate (mCPR) and demand for family planning satisfied by modern methods (mDFPS) in 48 countries as part of the Family Planning 2030 (FP2030) initiative between 1990 and 2020 for which Demographic and Health Survey data were available.

**Methods:**

We analysed two rounds of Demographic and Health Survey data per country. Changes in concentration indices between two survey rounds were compared to measure reductions in overall socioeconomic-related inequalities in modern contraceptive use. Poisson regression models were used to measure the adjusted average annual rate of change across wealth quintiles.

**Findings:**

In this population-based analysis study, all countries reduced socioeconomic-related inequalities in modern contraceptive use among in-union women of reproductive age (15–49 years) during the observed 30-year period. On average, mCPR increased at an annual rate of 2·1% (95% CI 2·1–2·2), and the rate of increase for the poorest women was 3·1% (3·0–3·2), which outpaced the rate of increase for the richest women of 1·3% (1·3–1·4%). The pattern of progress was similar for mDFPS, but at a slower pace. Overall, levels of mCPR and mDFPS increased, and socioeconomic-related inequalities were reduced during this period.

**Interpretation:**

Substantial progress has been made in reducing socioeconomic-related inequalities in family planning across the 48 studied countries, which account for 86% of the population of the 82 FP2030 initiative countries. During the past three decades, poorer women have seen greater improvements in modern contraceptive use and demand satisfaction compared with richer women. As contraceptive prevalence rates are near their maximum, it is crucial to ensure marginalised and vulnerable groups are not left behind.

**Funding:**

Bill & Melinda Gates Foundation.

**Translations:**

For the French and Spanish translations of the abstract see Supplementary Materials section.

## Introduction

Almost 30 years ago, at the International Conference on Population and Development, the importance of sexual and reproductive health and rights for sustainable development was emphasised. The International Conference on Population and Development Programme of Action called for a reduction in inequality and access to comprehensive reproductive health care and services, including voluntary family planning, for all people.^1^, ^2^, ^3^ Nearly 20 years later, at the London Summit on Family Planning in 2012, the family planning and development communities came together to establish a common goal: to provide 120 million women and girls with access to contraceptives and reproductive health services.^4^ This goal was operationalised through the creation of the Family Planning 2020 initiative, which has seen great strides in 71 economically disadvantaged countries with high family planning needs. Even though the target of 120 million additional users was not reached,^5^ 87 million additional users were added, and the number of women with unmet need for contraception decreased.^6^ The initiative continues as Family Planning 2030 (FP2030) to further improve reproductive health in low-income and middle-income countries (LMICs).

Despite global progress in access to contraception, the use of modern contraceptives by women at the bottom of the income distribution remains low and the gaps in use across wealth quintiles generally remain,^7^, ^8^, ^9^ which has been confirmed by more recent studies.^10^, ^11^, ^12^, ^13^, ^14^ A study by Ogundele and colleagues^15^ found that in Burkina Faso, Niger, Senegal, Nigeria, and Ghana, less wealthy women have characteristics beyond their ability to afford contraception—such as exposure to mass media messaging— that prevent them from accessing family planning services, compared with women in wealthier quintiles.

**Research in context**

**Evidence before this study**

Despite the commitments made by numerous countries at the 1994 International Conference on Population and Development and the 2012 London Summit on Family Planning and the interventions that followed, socioeconomic inequalities in contraceptive use have persisted. There have been, however, important achievements: the London Summit's goal of reaching 120 million additional users across 81 countries by 2020 was not met, but sub-Saharan Africa almost doubled the number of users of modern contraceptive methods between 2012 and 2022. But to what extent were these gains equitable? There have been analyses studying the socioeconomic inequalities in the demand for family planning satisfied by modern methods, and for vulnerable groups such as adolescent girls and young women, but there is a lack of comprehensive analysis that compares the progress in reducing socioeconomic inequalities across family planning indicators. We searched PubMed, Embase, and Google on June 21, 2024**,** using the search terms “socioeconomic inequalities”, “socioeconomic disparities”, “income inequality”, “wealth inequality”, “economic inequality”, “social inequality”, “family planning”, “contraception”, “modern contraceptive prevalence rate”, “mCPR”, “demand for family planning satisfied with modern methods”, “mDFPS”, “LMIC”, “low and middle-income countries”, “FP2030”, and “FP2020”.

**Added value of this study**

This study provides a comprehensive overview of the progress made between 1990 and 2020 in reducing socioeconomic inequalities in the use of modern contraceptives and in the demand for family planning satisfied by modern methods across 48 countries as part of the Family Planning 2020 (FP2020) initiative that, in 2012, pledged to increase the number of additional users of modern contraception by 2020. We also analysed a sample of seven countries that have collected data during the COVID-19 pandemic to assess whether the gains in reducing socioeconomic inequalities have been hampered by the pandemic. Our results suggest that virtually all countries in the FP2030 initiative included in our analysis reduced family planning inequalities across the income distribution among in-union women of reproductive age (15–49 years) during the last 30 years. However, gaps are still persistent and there is a need to identify interventions to bridge gaps in inequality and accelerate progress toward achieving equality in the use of contraception for preventing unwanted pregnancies.

**Implications of all the available evidence**

The findings from this study provide evidence of the progress made in reducing socioeconomic inequalities in family planning across 48 priority countries, which cover 86% of the population of the 82 Family Planning 2030 initiative countries. The FP2020 initiative covered 71 countries that included South Africa. The FP2030 initiative expanded to 81 countries and removed South Africa. We started this analysis with the FP2020 list of countries and updated it to include the countries included in the FP2030 refresh. Hence, we chose to keep South Africa in the analysis. During the last three decades, all 48 countries reduced socioeconomic-related inequalities in modern contraceptive use and in the demand for family planning satisfied by modern methods. Poorer women experienced higher average annual rates of change in modern contraceptive use and demand satisfied by modern methods compared to richer women and, thus, reduced the prevalence gaps. However, as countries approach the maximum achievable contraceptive prevalence rate, no one should be left behind, especially marginalised and vulnerable groups such as those at the bottom of the income distribution. Moreover, among the seven countries that collected data during the COVID-19 pandemic, we observed a decrease in the average annual rate of change in the use of contraception between the non-COVID-19-era and the COVID-19-era surveys in the poorest to richer wealth quintiles, despite that socioeconomic-related inequalities in the use of contraception were reduced. The findings from this study can be used by researchers, policy makers, and programme implementers in the FP2030 initiative priority countries for planning purposes. The methods can also be applied for performance monitoring and evaluating national and global initiatives such as the FP2030 initiative.

Differences in contraceptive use across the wealth distribution are well noted, but that is not to say these differences have remained stagnant in the past three decades. Increasingly, trends in this gap have been studied to determine where and how family planning efforts, such as the FP2030 initiative, should consider the future of contraceptive uptake and the demand being satisfied. One such study by Ross^9^ showed a mixed trend in the reduction of the rich–poor gap in the modern contraceptive prevalence rate (mCPR**)** in 46 countries globally from 1990 to 2013.^9^ Overall, the gap declined between the richest and poorest women, but in sub-Saharan Africa, the rich–poor gap increased in countries that had large income inequalities.^9^

Hellwig and colleagues^13^ examined trends in 73 LMICs from 1993 to 2017 and also found declines in wealth-related inequalities in the demand for family planning satisfied with modern methods (mDFPS).^13^ Evidence suggests that inequalities persist and have increased in some countries, namely in western and central Africa, but most of the countries saw much faster increases in mDFPS among poorer individuals, contributing to reductions in absolute and relative inequalities alike.^13^ This promising trend in reducing the rich–poor gap in mDFPS is also evident in the study by Mutua and colleagues,^11^ albeit for just 32 African countries analysed for available data since 2000. Nonetheless, the promise of such gap reductions is tempered by persistent inequalities between married and unmarried sexually active adolescent girls and young women.

Although the studies by Mutua and colleagues^11^ and Hellwig and colleagues^13^ are highly informative, they have limitations. These limitations were in various forms: focused on few countries with limited global scopes, covered a shorter time period to analyse trends,^11^ and restricted the analyses to women who were married or in-union.^13^ Strong efforts have been made since 2012 toward the achievement of the FP2020 initiative goals in the 71 target countries, but there is a dearth of literature examining how socioeconomic-related inequalities in contraceptive uptake have changed over time.

For this study, we used data from the first and most recent Demographic and Health Survey conducted across 48 focus countries that are part of the FP2030 initiative and aimed to measure the progress in reducing socioeconomic-related inequalities in mCPR and mDFPS among women of reproductive age between 1990 and 2020.

## Methods

### Study design and participants

This population-based analysis study uses data from nationally representative household-based surveys conducted by the Demographic and Health Survey Program. The Demographic and Health Survey collects data to monitor and evaluate population, health, and nutrition programmes.^16^ For this study, we used individual-level information about contraceptive behaviour and socioeconomic characteristics, both reported by women of reproductive age (15–49 years). To capture the household's cumulative standard of living and economic status, we used the household asset-based wealth quintile information.^17^

The Demographic and Health Survey has collected cross-sectional data for 59 countries that are part of the FP2030 initiative. A list of these countries along with the year of the first (Round-*1*) and most recent (Round-*n*)survey round, with the years available, have been provided in appendix 3 (pp 1–2). We required two survey rounds per country to measure progress in reducing socioeconomic-related inequalities in family planning. This restriction reduced our sample to 50 countries. In addition, Eritrea's database has restricted access and Yemen did not collect wealth index data in 1991–92, which further reduced our sample to 48 countries that have had at least two Demographic and Health Survey rounds at any point in time. Our analysis included 31 countries from sub-Saharan Africa, two countries from the Middle East and north Africa, five countries from east Asia and the Pacific, four countries from south Asia, four countries from Latin America and the Caribbean, and two countries from Europe and central Asia. Surveys go back as far as 1990 for Nigeria and Pakistan, and the most recent surveys were collected between 2019 and 2020 in Sierra Leone, Senegal, Liberia, The Gambia, and Rwanda.

The analysis presented in this study is limited to women who, at the time of the interview, indicated they were either married or living with a partner to be consistent with the FP2030 initiative that tracks mDFPS only for in-union women, but we present complementary results for all women in appendix 3 (pp 11–14). Demographic and Health Survey surveys aimed to recruit women aged 15–49 years and children aged younger than 5 years who live in residential households. Many surveys also included men aged 15–59 years. However, the specific target population can differ depending on the country or the survey.^18^

All surveys used for this analysis included de-identified publicly available data. Ethical approval was exempted for this analysis of secondary data.

### Procedures

To capture contraceptive behaviour (eg, the decision to use contraception and the decision to select a specific method of contraception), we used mCPR and mDFPS indicators as our outcomes of interest for women of reproductive age. Modern contraceptive methods were oral pills, intrauterine devices, injections, female or male condoms, female or male sterilisation, implants or Norplants, and lactational amenorrhea.^16^ mCPR was constructed as a binary indicator, taking the value of 1 if a woman reported she was using a modern method of contraception at the time of the interview or if she reported that her partner was using one, but otherwise 0 was used.^19^ Women included in the mCPR calculation are all women of reproductive age. mDFPS is a binary variable that takes the value of 1 if a woman is using modern contraception given that she has a demand for family planning and takes the value of 0 if a woman is not using modern contraception given that she has a demand for family planning. Women included in the mDFPS calculation are all women of reproductive age who demand family planning methods, defined as those who either have an unmet need for family planning or are currently using any family planning method. From here on, we will use demand for family planning satisfied with modern methods and demand satisfied interchangeably or refer to this variable as mDFPS.

Household wealth was proxied by linear index of asset ownership indicators using a principal components analysis,^17^ which is constructed by the Demographic and Health Survey program and grouped into a quintile index. The wealth index is a composite measure based on a set of selected assets, which is country specific, that reflect a household's ownership of several consumer items related to wealth status. Such items are ownership of a television and car, dwelling characteristics such as flooring material, type of drinking water source, toilet facilities, and other characteristics.^20^ Empirical evidence has shown that the wealth index, also known as the asset index, is highly correlated with income and is a good proxy for socioeconomic status.^17^

### Statistical analysis

To measure the progress of the country in the reduction of relative inequality in the use of modern contraceptives and in the demand for family planning satisfied by modern methods over the distribution of household wealth, we first computed a concentration index, which is a common measure in health economics analogous to the Gini index to assess socioeconomic-related inequality, separately for each country.^21^, ^22^ The cumulative proportion of women using modern contraceptives (or the proportion of women with a demand for family planning satisfied by modern methods) can be plotted against the cumulative proportion of women ranked by household wealth. This plot is known as the concentration curve, which is analogous to the Lorenz curve that shows income inequalities.^23^ If equality exists, the line should have a 45° angle; however, the curve will lie above or below the 45° line when inequalities are present. appendix 3 (p 3) illustrates an example of two extreme cases for mCPR. The concentration curve in Bolivia lies below the 45° line, which means that no use of contraceptives is heavily concentrated among the poorest individuals, even though the country made substantial progress between 1994 and 2008. A contrary example is the Philippines where inequalities in 2017 were heavily concentrated among the richest women. The concentration index is twice the area between the 45° line and the concentration curve. Modern contraceptive prevalence rate is a bounded binary variable that takes the value of 0 if a woman (*i*) is not using a contraceptive method (or has an unsatisfied demand for family planning) at time (*t*), and equals to 1 if a woman (*i*) is using a modern contraceptive method (or has a satisfied demand for family planning) at time (*t*). Given that our variable of interest is binary and the level of measurement is fixed—meaning that 0 corresponds to a situation of complete absence^21^—we computed the standard concentration index as:

$$C\left(h|y\right)=\frac{2\mathrm{cov}\left(h_{1},R_{1}\right)}{\bar{h}}=\frac{1}{n}\sum_{i=1}^{n}\left[\frac{h_{i}}{\bar{h}}\left(2R_{i}-1\right)\right]$$

where (2*R*_i_ – 1) represents the household wealth rank and *h*_i_ the use of modern contraceptives (or demand satisfied for family planning) of the woman (*i*)*. C* ranges between (1 – *I*)/*I*, when maximal pro-rich inequality exists, to (1 – *I*)/*I*, when maximal pro-poor inequality exists. Indices were computed separately for the first and most recent Demographic and Health Survey round conducted in the country *j* (*j*=1, …, 48).

We specified a Poisson regression model to measure the adjusted average annual rate of change^24^ in mCPR and mDFPS among married women of reproductive age between the first and most recent Demographic and Health Survey round. To do so, we pooled the two survey rounds per country and estimated our regressions separately for each country with individual-level data. Our model was specified as:

log(y_ij_)=a + y_j_S Round_j_ + β_j_X_ij_ + ɛ_ij_ for Wealth={poorest, 2, 3, 4, richest}

where y_ij_ represents the probability that a woman *i*: (*i*=1, …, *I*) in the country (*j*) uses a modern contraceptive or has a demand for family planning satisfied by modern methods. Each outcome is specified separately. The model regresses (y_ij_) on the year when surveys were conducted (SRound_j_), and demographic characteristics (X_ij_), stratified by wealth quintiles. Demographic characteristics were age and parity, both measured as continuous variables ranging from 15 to 49 for age and from 0 to 21 for the number of children, and an indicator variable measuring the area of residence (urban or rural). ɛ_ij_ represents the error term. Our parameter of interest is y_j_, which measures the adjusted average annual rate for modern contraceptive use and demand satisfied in each survey round by wealth quintile. We also estimated unadjusted average annual rates of change as the

$$\log\left(\frac{{\text{Outcome}}_{\text{Round}1}}{{\text{Outcome}}_{\text{Round}n}}/\text{years}\;\text{between}\;\text{surveys}\right).$$

### Role of the funding source

The funder of the study had no role in study design, data collection, data analysis, data interpretation, or writing of the report.

## Results

**Figure 1:**
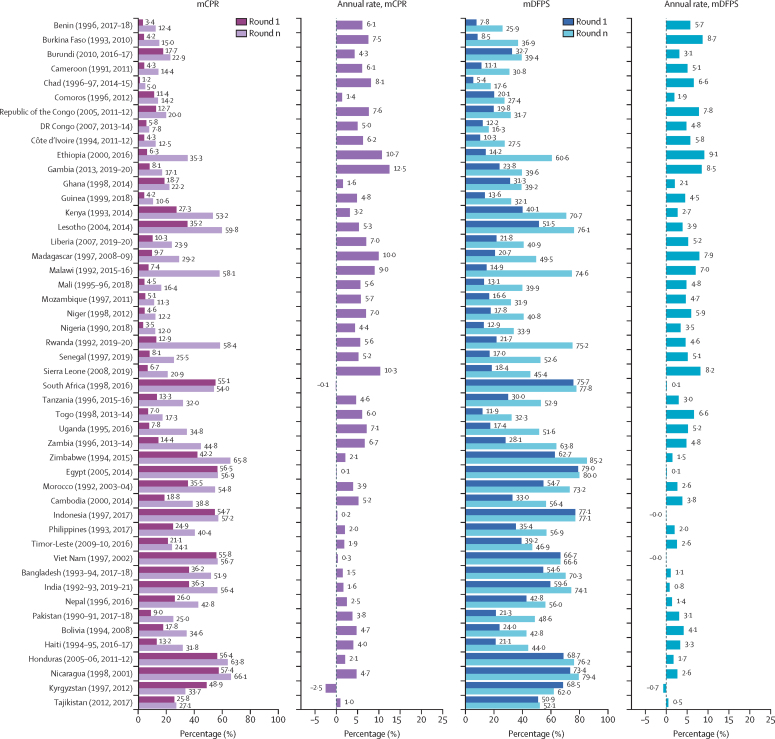
Changes in mCPR and mDFPS between the first and most recent Demographic and Health Survey round in in-union women Country estimates for modern contraceptive prevalence rate (mCPR) and family planning satisfied by modern methods (mDFPS) have been shown in appendix 3 (p 4).

For more than 30 years, all countries included in our analysis, except for Kyrgyzstan and South Africa, experienced an increase in mCPR and mDFPS among in-union women of reproductive age (figure 1). Malawi stood out, increasing its mCPR by 50·7 percentage points and mDFPS by 59·7 percentage points in 24 years. Gambia, Ethiopia, and Sierra Leone registered the fastest pace of increase in both outcomes of interest, with an average annual rate of 12·5%, 10·7%, and 10·3% for mCPR and 8·5%, 9·1%, and 8·2% for mDFPS for more than 7 years, 16 years, and 11 years, respectively.

**Figure 2:**
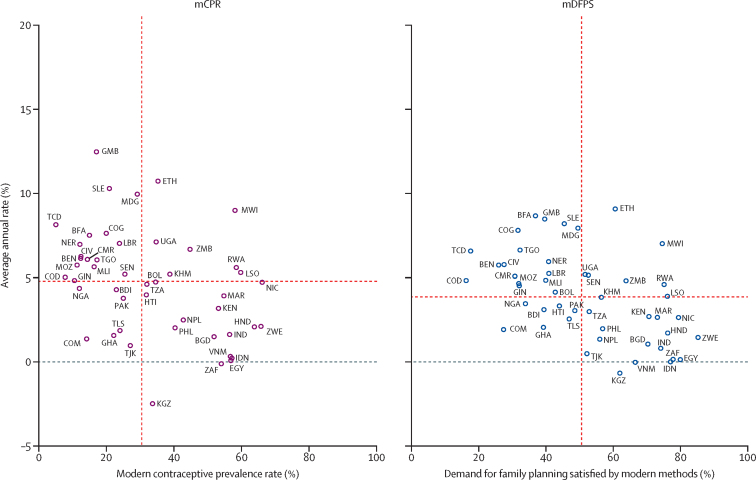
mCPR and mDFPS levels and average annual rates of change stratified by their median value for in-union women The red dotted lines represent the median value. BDI=Burundi. BEN=Benin. BFA=Burkina Faso. BGD=Bangladesh. BOL=Bolivia. CIV=Côte d’Ivoire. CMR=Cameroon. COD=Democratic Republic of the Congo. COG=Republic of the Congo. COM=Comoros. EGY=Egypt. ETH=Ethiopia. GHA=Ghana. GIN=Guinea. GMB=Gambia. HND=Honduras. HTI=Haiti. IDN=Indonesia. IND=India. KEN=Kenya. KGZ=Kyrgyzstan. KHM=Cambodia. LBR=Liberia. LSO=Lesotho. MAR=Morocco. mCPR=modern contraceptive prevalence rate. mDFPS=family planning satisfied by modern methods. MDG=Madagascar. MLI=Mali. MOZ=Mozambique. MWI=Malawi. NER=Niger. NGA=Nigeria. NIC=Nicaragua. NPL=Nepal. PAK=Pakistan. PHL=Philippines. RWA=Rwanda. SEN=Senegal. SLE=Sierra Leone. TCD=Chad. TGO=Togo. TJK=Tajikistan. TLS=Timor-Leste. TZA=Tanzania. UGA=Uganda. VNM=Vietnam. ZAF=South Africa. ZMB=Zambia. ZWE=Zimbabwe.

Figure 2 shows countries’ distribution across their most recent level of mCPR and mDFPS and their corresponding average annual rate of progress. We used the median value of these rates as a threshold to divide countries into four quadrants. The first quadrant groups countries with rates above the median, and countries in the third quadrant groups countries with rates below the median. The second quadrant groups countries with an mCPR (or mDFPS) below the median and its corresponding average annual rate above the median. The fourth quadrant complements the second quadrant. Malawi and Ethiopia were clear outliers in the first quadrant. Similar outliers were Gambia, Sierra Leone, Chad, and the Democratic Republic of the Congo in the second quadrant, Comoros and Ghana in the third quadrant, and Kyrgyzstan, Zimbabwe, Egypt, and Viet Nam in the fourth quadrant.

**Figure 3:**
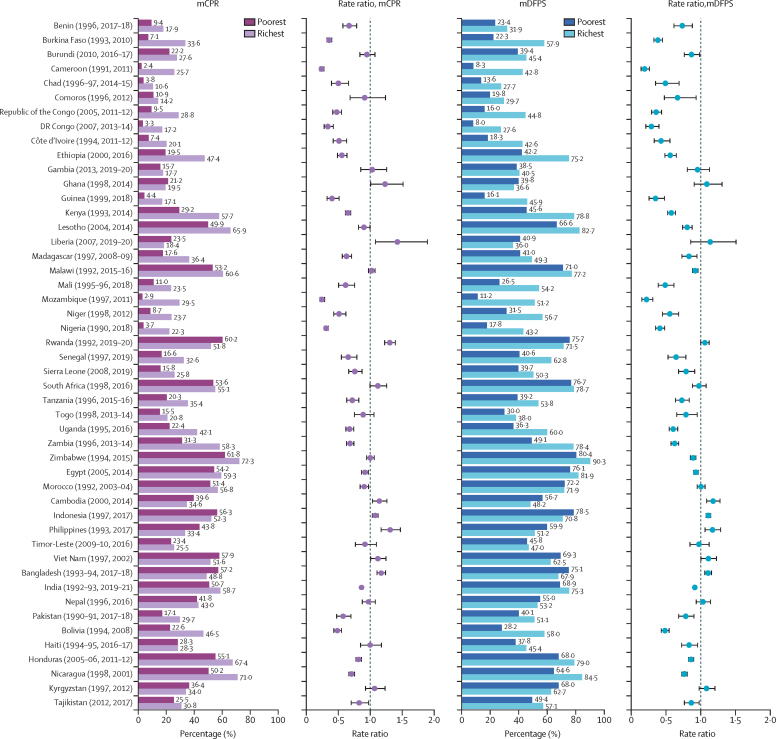
Differences in mCPR and mDFPS between the richest and poorest wealth quintiles in the most recent Demographic and Health Survey round for in-union women Poorest and richest ratios were not adjusted for demographic characteristics. Country estimates have been shown in appendix 3 (pp 5–6). mCPR=modern contraceptive prevalence rate. mDFPS=family planning satisfied by modern methods.

Differences in mCPR and mDFPS between in-union women from the richest and poorest wealth quintiles using the most recent Demographic and Health Survey round are presented in figure 3. A ratio of 1 means that the proportion of contraceptive users (or women with a satisfied demand) among the poorest women is equivalent to those in the richest wealth quintile. A ratio above 1 indicates that women from the poorest wealth quintile have a higher rate than women from the richest wealth quintile; a ratio below 1 indicates the contrary. Among the 48 FP2030 initiative study countries, nine countries recorded no significant differences in mCPR between the poorest and richest women, which were Haiti, South Africa, Nepal, Ghana, Gambia, Kyrgyzstan, Timor-Leste, Bangladesh, and Comoros, and among them, Comoros, Ghana, Gambia, and Timor-Leste had an mCPR below 30%. In Bolivia (2008), Madagascar (2008–09), Mali (2018), Ethiopia (2016), Niger (2012), Côte d’Ivoire (2011–12), Chad (2014–15), Republic of the Congo (2011–12), Guinea (2018), Burkina Faso (2010), Democratic Republic of the Congo (2013–14), Nigeria (2018), Mozambique (2011), and Cameroon (2011), women from the poorest wealth quintile had an mCPR that was less than half of women's mCPR from the richest wealth quintile. As for mDFPS, only eight countries did not have significant differences between the poorest and richest women, which were Gambia (2019–20), Ghana (2014), Liberia (2019–20), South Africa (2016), Morocco (2003–04), Timor-Leste (2016), Nepal (2016), and Kyrgyzstan (2012). Rwanda (2019–20), Cambodia (2014), Indonesia (2017), the Philippines (2017), Viet Nam (2002), and Bangladesh (2017–18) had a significant ratio above 1 in both mCPR and mDFPS.

**Figure 4:**
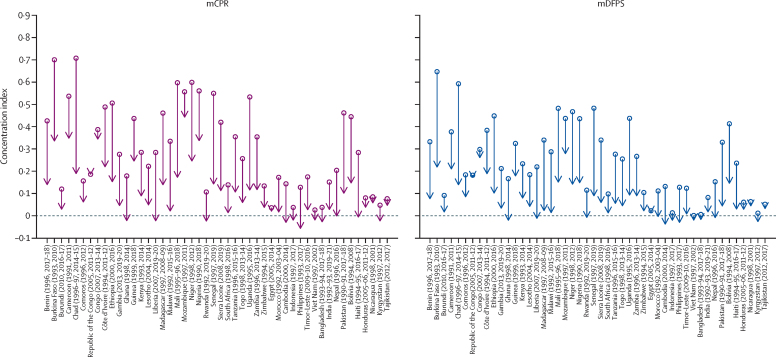
Changes in concentration indices for mCPR and mDFPS between the first and most recent Demographic and Health Survey round for in-union women Country estimates for modern contraceptive prevalence rate (mCPR) and family planning satisfied by modern methods (mDFPS) have been shown in appendix 3 (p 7).

Concentration indices computed for all countries and survey rounds have been shown in figure 4. The head of the arrow represents the concentration index computed for the most recent Demographic and Health Survey, whereas the tail end of the arrow represents the first survey. The index is bounded between –1 and 1; an index of 0 represents perfect equality, a positive index indicates pro-poor inequalities, and a negative index indicates pro-rich inequalities. If the health variable is a good outcome, such as the use of contraception, a positive value of the concentration index means the use of contraception is lower among poor individuals (pro-poor) compared with rich individuals, and a negative value of the concentration index means the outcome is higher among rich individuals (pro-rich) compared with poor individuals. All countries had a positive index in their first Demographic and Health Survey round for both mCPR and mDFPS, which means that, historically, contraceptive use was concentrated among the richest women. Figure 4 also shows that all countries experienced a reduction in socioeconomic-related inequalities in mCPR and mDFPS among in-union women. The top ten countries with the greatest reduction in mCPR inequalities across wealth distribution were Chad (0·52 point reduction), followed by Mali (0·43), Uganda (0·42), Senegal (0·41), Burkina Faso (0·37), Pakistan (0·36), Niger (0·35), Ethiopia (0·35), Madagascar (0·32), and Malawi (0·31). The list of top ten performers in reducing inequalities in mDFPS across the wealth distribution is the same as for the mCPR, except Nigeria substitutes Malawi in the list.

**Figure 5:**
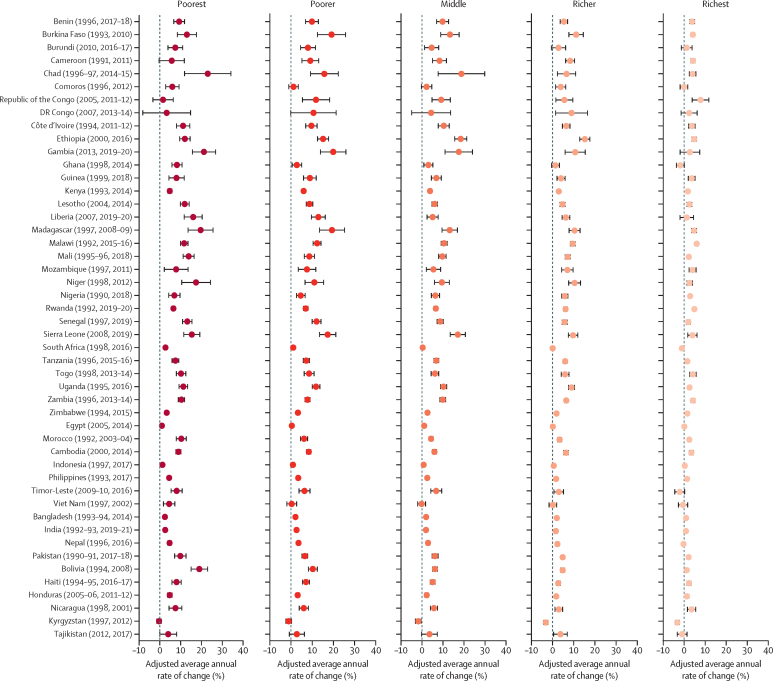
Adjusted average annual rate of change in mCPR between the first and most recent Demographic and Health Survey for in-union women by wealth quintile Country estimates for the modern contraceptive prevalence rate (mCPR) have been shown in appendix 3 (p 8).

**Figure 6:**
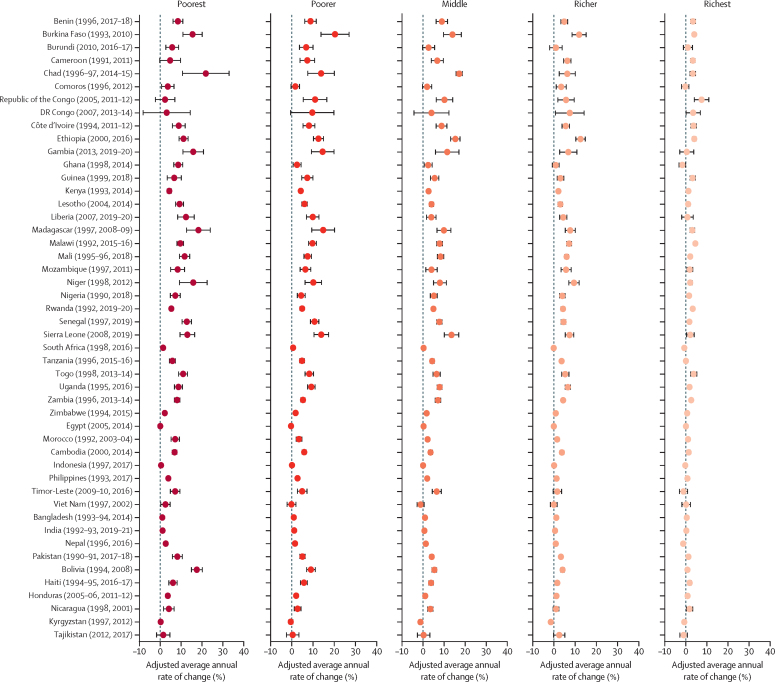
Adjusted average annual rate of change in mDFPS between the first and most recent Demographic and Health Survey for in-union women by wealth quintile Country estimates for family planning satisfied by modern methods (mDFPS) have been shown in appendix 3 (p 9).

Figure 5 shows the adjusted average annual rate of change between the first and most recent Demographic and Health Survey round stratified by wealth quintiles for mCPR, and figure 6 shows the same, but for mDFPS. On average, in-union women increased their mCPR at an annual rate of 2·1% (95% CI 2·1–2·2), and the rate of increase for the poorest women was 3·1% (3·0–3·2), outpacing the rate of increase for the richest women of 1·3% (1·3–1·4). This pattern was observed across all countries except in Republic of the Congo. For mDFPS, the average annual rate of change was 1·0% (95% CI 0·9–1·0) for all in-union women, whereas it was 1·6% (1·5–1·7) for the poorest women compared with 0·6% (0·5–0·6) for the richest women. This pattern held in all countries except in Egypt, the Democratic Republic of the Congo, and Republic of the Congo. These findings suggest that even though women from the poorest wealth quintile started with a lower level of contraceptive use, they were able to increase their use to catch up with the women in higher economic quintiles and satisfy their demand at a faster pace than women from the highest wealth quintile.

This study could not assess the effects of the COVID-19 pandemic on the use of contraception due to data limitations. However, we conducted a separate analysis with data collected between 2021 and 2022, which was available for seven countries. These countries were Cambodia (2021–22), Côte d’Ivoire (2021), Kenya (2022), Madagascar (2021), Nepal (2022), Philippines (2022), and Burkina Faso (2021). We repeated our analysis to measure progress in reducing socioeconomic-related inequalities in the mCPR and mDFPS using these surveys as the most recent survey round (appendix 3 p 10), and our conclusions did not change. Among these seven countries, the average annual rate of change in mCPR for women in the poorest wealth quintile was 4·3% (95% CI 4·0–4·7) compared with 2·0% (1·8–2·2) among the richest women. The pattern in mDFPS was similar, but of smaller magnitude—a 2·5 percentage points difference between the poorest and richest wealth quintiles. However, the pace of improvement between the first survey round and the COVID-19-era survey was slower when compared with the pace of improvement calculated with the non-COVID-19-era survey across all of these countries and wealth quintiles, with a few exceptions for women in the richest wealth quintile.

## Discussion

All countries in the FP2030 initiative that were included in our analysis reduced family planning inequalities across the income distribution among in-union women of reproductive age during the last 30 years. Nonetheless, in nearly all countries, we found persistently lower mCPRs among poorer women versus richer women, except in nine countries. Despite this pattern in all countries, poorer women observed higher average annual rates of change in modern contraceptive use and demand satisfaction compared with richer women, except in Republic of the Congo.

The results of this study are supported by and expanded upon extant literature, such as the study by Ross^9^ that found a decline in the rich–poor gap in mCPR; however, this was tempered by an increase in the gap for some sub-Saharan African countries. This increase in the mCPR gap was not found in our selection of countries. More recent studies have focused on mDFPS; Hellwig and colleagues^13^ and Mutua and colleagues^11^ both showed promising declines in wealth-related inequalities in mDFPS, which is confirmed by our study. Although Hellwig and colleagues^13^ found increases in inequalities among a few countries, for example, in west and central Africa, our results have not echoed the same results. Given the varying scopes and measurement approaches of these past investigations, this study has expanded on previous evidence for both the mCPR and mDFPS with a targeted examination of FP2030 initiative target countries to capitalise on ongoing efforts in these nations.

There are several strengths to this study. First and foremost, we have presented evidence of progress in reducing socioeconomic inequalities in both mCPR and mDFPS, showing a comprehensive overview of change over time. By investigating both of these measures, we inform a wider context of family planning efforts to, directly and indirectly, address family planning needs within and across countries, which further speaks to a broader audience of researchers, practitioners, and policy makers in target countries as part of the FP2030 initiative. Additionally, we assessed inequalities by looking at change over the entirety of the income distribution and the two extreme quintiles of the income distribution. This dual approach provides a more nuanced examination of how trends changed within countries to further reduce any ways that aggregate measures mask in-country patterns. Finally, this study investigated progress during 30 years, which is a wide range of time that captures the earliest and some of the most recent efforts to improve contraceptive uptake and overall wellbeing.

This study is not without limitations. First, due to the use of Demographic and Health Survey data, we used a proxy of income that is measured at the household level, which does not allow us to directly estimate a woman's income or allow us to see how it is linked to her contraceptive use behaviour. Second, we were unable to include all countries included in the FP2030 initiative because our analysis approach required there to be at least two Demographic and Health Survey surveys per country. Third, all countries included in our analysis improved their socioeconomic conditions from 1990; the smallest exponential growth in GDP per capita was in Burundi from US$206 in 1990 to $217, and the largest by Viet Nam from $97 in 1990 to $3586. No GDP data were available for Timor-Leste, Mozambique, Liberia, and Cambodia in 1990.^25^ The improvements in mCPR and mDFPS we recorded in our analysis could be influenced by these improvements in overall socioeconomic conditions, and we cannot disentangle their contribution nor know whether they benefited women from all income strata; nonetheless, our estimated annual growth rates in mCPR and mDFPS were controlled by the level of wealth in the household. Fourth, we limited the analysis to women who are married or in-union to align with the FP2030 initiative core indicator for mDFPS, which is also limited to this group. However, we expanded our analysis of socioeconomic inequalities to all women of reproductive age (appendix 3 pp 11–14). Finally, with our results, we cannot draw conclusions or provide interpretations at the regional level given that, outside of sub-Saharan Africa, the regions included in our analysis have a small sample of countries.

To overcome the described limitations, future research should focus on expanding the scope of the FP2030 initiative target countries to ensure adequate sample size within regions, although this depends on the continuation of the surveys by the Demographic and Health Survey and the potential of leveraging data from other national-level surveys, such as the Multiple Indicator Cluster Surveys. Moreover, the COVID-19 pandemic negatively affected household income and food security,^26^ and even though in some instances it did not increase income inequality,^27^ it is important to continue tracking the effect of the COVID-19 pandemic on sexual and reproductive health outcomes. Most of these issues are strongly linked to measurement approaches that are inherent to extant data collection methods, and so the research community should consider how to overcome these challenges from the conception of data collection and definition of indicators through the analytical process.

This study has shown that, during the last 30 years, there has been a reduction in socioeconomic-related inequalities in family planning across 48 of the target countries of the FP2030 initiative. In all countries, poorer women compared with their richer counterparts experienced a higher annual rate of change in both modern contraceptive use and the demand for family planning satisfied by modern methods, except in Republic of the Congo. The progress is remarkable; nonetheless, countries are beginning to approach the maximum achievable contraceptive prevalence rate, and so family planning and development communities must continue to endeavour to leave no person behind.

## Contributors

## Data sharing

Data collected for this study are publicly available on the website of the Demographic and Health Survey Program at https://dhsprogram.com/data/available-datasets.cfm.

## Declaration of interests

We declare no competing interests.

## Supplementary Materials

French translation of the abstract

Spanish translation of the abstract
