# Supplementary material for: Progress in reducing socioeconomic inequalities in the use of modern contraceptives in 48 focus countries as part of the FP2030 initiative between 1990 and 2020: a population-based analysis
Source: Lancet Glob Health. 2024 Dec 18;13(1):e38–49. doi: 10.1016/S2214-109X(24)00424-8 (PMC11659844; doi:10.1016/S2214-109X(24)00424-8)
Supplement: French translation of the abstract [file mmc1.pdf]

THE LANCET
Global Health

Supplementary appendix 1

This translation in French was submitted by the authors and we reproduce it as supplied. It has not been peer reviewed. *The Lancet*'s editorial processes have only been applied to the original in English, which should serve as reference for this manuscript.

Cette traduction en français a été proposée par les auteurs et nous l'avons reproduite telle quelle. Elle n'a pas été examinée par des pairs. Les processus éditoriaux du *Lancet* n'ont été appliqués qu'à l'original en anglais et c'est cette version qui doit servir de référence pour ce manuscrit.

Supplement to: Cardona C, Rusatira JC, Salmeron C, et al. Progress in reducing socioeconomic inequalities in the use of modern contraceptives in 48 focus countries as part of the FP2030 initiative between 1990 and 2020: a population-based analysis. *Lancet Glob Health* 2025; **13:** e38–49.

Resumé

Contexte: Malgré l'augmentation de l'utilisation des contraceptifs modernes, les inégalités socio-économiques en matière de planification familiale persistent. Dans cette étude, nous avons tenté à mesurer les progrès accomplis dans la réduction des inégalités socio-économiques en matière de taux de prévalence de la contraception moderne (TPCm) et de demande de planification familiale satisfaite par des méthodes modernes (DSm) dans 48 pays dans le cadre de l'initiative FP2030 entre 1990 et 2020 pour lesquels des données de l'enquête démographique et de santé étaient disponibles.

Méthodes: Nous avons analysé deux séries de données de l'enquête démographique et de santé par pays. Les changements dans les indices de concentration entre les deux séries d'enquêtes ont été comparés pour mesurer les réductions des inégalités socio-économiques globales dans l'utilisation des contraceptifs modernes. Les modèles de régression de Poisson ont été utilisés pour mesurer le taux annuel moyen de changement ajusté entre les quintiles de richesse.

Résultats: Dans cette étude d'analyse basée sur la population, au cours de la période de 30 ans étudiée, tous les pays ont réduit les inégalités socio-économiques en matière d'utilisation de contraceptifs modernes parmi les femmes mariées en âge de procréer (15-49 ans). En moyenne, le TPCm a augmenté à un taux annuel de 2,1% (IC 95% 2,1% - 2,2%), et le taux d'augmentation pour les femmes les plus pauvres était de 3,1% (3,0% – 3,2%), ce qui dépassait le taux d'augmentation pour les femmes les plus riches de 1,3% (1,3% – 1,4%) Les progrès ont été similaires pour le DFSPm, mais à un rythme plus lent Dans l'ensemble, les niveaux de TPCm et de DSm ont augmenté et les inégalités socio-économiques ont été réduites au cours de cette période. Dans cette étude d'analyse basée sur la population, tous les pays ont réduit les inégalités socio-économiques en matière d'utilisation de contraceptifs modernes chez les femmes en union qui étaient en âge de procréer (15-49 ans) au cours de la période étudiée de 30 ans. Les progrès ont été similaires pour le DSm, mais à un rythme plus lent. De façon générale, les niveaux de TPCm et de DSm ont augmenté et les inégalités socio-économiques ont été réduites au cours de cette période.

Interprétation: Des progrès substantiels ont été accomplis dans la réduction des inégalités socio-économiques en matière de planification familiale dans les 48 pays étudiés, qui représentent 86% de la population des 82 pays de l'initiative FP2030. Au cours des trois dernières décennies, les femmes les plus pauvres ont connu de plus grandes améliorations dans l'utilisation des contraceptifs modernes et la satisfaction de la demande par rapport aux femmes les plus riches. Les taux de prévalence contraceptive étant proches de leur maximum, il est essentiel de veiller à ce que les groupes marginalisés et vulnérables ne soient pas laissés en arrière.

Financements: Fondation Bill & Melinda Gates.

This translation in French was submitted by the authors and we reproduce it as supplied. It has not been peer reviewed. The Lancet's editorial processes have only been applied to the original in English, which should serve as reference for this manuscript.
