# Supplementary material for: Progress in reducing socioeconomic inequalities in the use of modern contraceptives in 48 focus countries as part of the FP2030 initiative between 1990 and 2020: a population-based analysis
Source: Lancet Glob Health. 2024 Dec 18;13(1):e38–49. doi: 10.1016/S2214-109X(24)00424-8 (PMC11659844; doi:10.1016/S2214-109X(24)00424-8)
Supplement: Spanish translation of the abstract [file mmc2.pdf]

THE LANCET
Global Health

Supplementary appendix 2

This translation in Spanish was submitted by the authors and we reproduce it as supplied. It has not been peer reviewed. *The Lancet*'s editorial processes have only been applied to the original in English, which should serve as reference for this manuscript.

Los autores nos proporcionaron esta traducción al español y la reproducimos tal como nos fue entregada. No la hemos revisado. Los procesos editoriales de *The Lancet* se han aplicado únicamente al original en inglés, que debe servir de referencia para este manuscrito.

Supplement to: Cardona C, Rusatira JC, Salmeron C, et al. Progress in reducing socioeconomic inequalities in the use of modern contraceptives in 48 focus countries as part of the FP2030 initiative between 1990 and 2020: a population-based analysis. *Lancet Glob Health* 2025; **13:** e38–49.

Resumen

Antecedentes: A pesar del aumento del uso de métodos anticonceptivos modernos, aún persisten las desigualdades socioeconómicas en la planificación familiar. En este estudio, nos propusimos medir los avances en la reducción de las desigualdades socioeconómicas en la tasa de prevalencia de uso de anticonceptivos modernos (PAM) y la demanda de planificación familiar satisfecha con métodos anticonceptivos modernos (DPFM) en 48 países que forman parte de la iniciativa FP2030 entre 1990 y 2020 para los que se disponía de datos de las Encuestas de Demografía y Salud.

Métodos: Para cada país, analizamos dos rondas de datos de las Encuestas de Demografía y Salud. Comparamos los cambios en los índices de concentración entre dos rondas de encuestas para medir la reducción de las desigualdades socioeconómicas generales en el uso de anticonceptivos modernos. Utilizamos modelos de regresión de Poisson para medir la tasa de crecimiento anual promedio ajustada para cada quintil de riqueza entre dos rondas de encuestas.

Resultados: En este estudio, todos los países redujeron las desigualdades socioeconómicas en el uso de anticonceptivos modernos entre las mujeres en edad reproductiva (15-49 años) durante el período observado de 30 años. En promedio, la PAM aumentó a una tasa anual de 2.1% (IC 95%: 2.1-2.2), y la tasa de aumento para las mujeres más pobres fue de 3.1% (3.0-3.2), que superó la tasa de aumento para las mujeres más ricas de 1.3% (IC 95%: 1.3-1.4%). El patrón de progreso fue similar para la DPFM, pero a un ritmo más lento. En general, los niveles de PAM y DPFM aumentaron, y las desigualdades socioeconómicas se redujeron durante este periodo.

Interpretación: Se han logrado avances sustanciales en la reducción de las desigualdades socioeconómicas relacionadas con la planificación familiar en los 48 países estudiados, que representan el 86% de la población de los 82 países de la iniciativa FP2030. Durante las últimas tres décadas, las mujeres más pobres han experimentado mayores mejoras en el uso de anticonceptivos modernos y en la satisfacción de la demanda en comparación con las mujeres más ricas. A medida que las tasas de prevalencia del uso de anticonceptivos se acercan a su máximo, es crucial garantizar que los grupos marginados y vulnerables no se queden atrás.

Financiación: Fundación Bill & Melinda Gates.

This translation in Spanish was submitted by the authors and we reproduce it as supplied. It has not been peer reviewed. The Lancet's editorial processes have only been applied to the original in English, which should serve as reference for this manuscript.
