# Supplementary material for: Progress in reducing socioeconomic inequalities in the use of modern contraceptives in 48 focus countries as part of the FP2030 initiative between 1990 and 2020: a population-based analysis
Source: Lancet Glob Health. 2024 Dec 18;13(1):e38–49. doi: 10.1016/S2214-109X(24)00424-8 (PMC11659844; doi:10.1016/S2214-109X(24)00424-8)
Supplement: Supplementary appendix 3 [file mmc3.pdf]

THE LANCET
Global Health

Supplementary appendix 3

This appendix formed part of the original submission and has been peer reviewed. We post it as supplied by the authors.

Supplement to: Cardona C, Rusatira JC, Salmeron C, et al. Progress in reducing socioeconomic inequalities in the use of modern contraceptives in 48 focus countries as part of the FP2030 initiative between 1990 and 2020: a population-based analysis. *Lancet Glob Health* 2025; **13:** e38–49.

Appendices

Appendix A: in-union women of reproductive age 1

Table A- 1: List of countries part of the FP2030 initiative that have had at least one DHS survey round conducted before 2020 1

Figure A- 1: Concentration Curve for Bolivia and the Philippines, in-union women 3

Table A- 2: mCPR and mDFPS reported in the first and most recent DHS round 4

Table A- 3: mCPR and mDFPS in the richest and poorest wealth quintiles in the most recent DHS round, in-union women 5

Table A- 4: Concentration indices (CI) for mCPR and mDFPS in the first and most recent DHS, in-union women 7

Table A- 5: Adjusted average annual rate of change in mCPR between the first and most recent DHS round, in-union women, by wealth quintile 8

Table A- 6: Adjusted average annual rate of change in mDFPS between the first and most recent DHS round, in-union women, by wealth quintile 9

Table A- 7: Average annual rate of change in mCPR and mDFPS between the first and most recent DHS collected during the COVID-19 pandemic by wealth quintiles, and concentration indices, in-union women 10

Appendix B: all women of reproductive age **11**

Table B- 1: mCPR and mDFPS reported in the first and most recent DHS round, all women 11

Table B- 2: mCPR and mDFPS in the richest and poorest wealth quintiles in the most recent DHS round, all women 12

Table B- 3: Concentration indices (CI) for mCPR and mDFPS in the first and most recent DHS, all women 14

Appendix A: in-union women of reproductive age

Table A- 1: List of countries part of the FP2030 initiative that have had at least one DHS survey round conducted before 2020

Country	ISO3 Alpha-code	Year of Survey Round-*1*	Year of Survey Round-*n*	Included in Analysis	Population
**Sub-Saharan Africa**					
Angola	AGO	n.a.	n.a.	No	33,958.0
Benin	BEN	1996	2017-18	Yes	12,819.4
Burkina Faso	BFA	1993	2010	Yes	21,813.4
Burundi	BDI	2010	2016-17	Yes	12,386.6
Cabo Verde	CPV	n.a.	n.a.	No	585.3
Cameroon	CMR	1991	2011	Yes	26,845.0
Central African Rep.	CAF	n.a.	1994-95	No	5,414.0
Chad	TCD	1996-97	2014-15	Yes	16,910.2
Comoros	COM	1996	2012	Yes	814.0
Congo	COG	2005	2011-12	Yes	5,769.1
Congo Dem. Rep.	COD	2007	2013-14	Yes	94,374.4
Côte d'Ivoire	CIV	1994	2011-12	Yes	27,146.0
Eritrea	ERI	1995	2002	No	3,588.1
Eswatini	SWZ	n.a.	n.a.	No	1,187.1
Ethiopia	ETH	2000	2016	Yes	118,743.5
Gambia	GMB	2013	2019-20	Yes	2,606.9
Ghana	GHA	2003	2014	Yes	32,511.6
Guinea	GIN	1999	2018	Yes	13,368.7
Guinea-Bissau	GNB	n.a.	n.a.	No	2,038.4
Kenya	KEN	1993	2014	Yes	52,511.3
Lesotho	LSO	2004	2014	Yes	2,268.6
Liberia	LBR	2007	2019-20	Yes	5,141.0
Madagascar	MDG	1992	2008-09	Yes	28,571.2
Malawi	MWI	1992	2015-16	Yes	19,633.2
Mali	MLI	1995-96	2018	Yes	21,561.3
Mauritania	MRT	n.a.	2000-01	No	4,556.3
Mozambique	MOZ	1997	2011	Yes	31,635.7
Niger	NER	1992	2012	Yes	24,785.6
Nigeria	NGA	1990	2018	Yes	210,874.2
Rwanda	RWA	1992	2019-20	Yes	13,305.4
Sao Tome and Principe	STP	n.a.	2008-09	No	221.1
Senegal	SEN	1997	2019	Yes	16,656.8
Sierra Leone	SLE	2008	2019	Yes	8,327.7
Somalia	SOM	n.a.	n.a.	No	16,801.2
South Africa	ZAF	1998	2016	Yes	59,137.7
South Sudan	SSD	n.a.	n.a.	No	10,667.0
Sudan	SDN	1989-90	n.a.	No	45,052.9
Tanzania	TZA	1991-92	2015-16	Yes	62,637.1
Togo	TGO	1998	2013-14	Yes	8,542.9
Uganda	UGA	1995	2016	Yes	45,123.4
Zambia	ZMB	1992	2013-14	Yes	19,200.5
Zimbabwe	ZWE	1994	2015	Yes	15,834.1
**Middle East and North Africa**					
Algeria	DZA	n.a.	n.a.	No	43,812.3
Djibouti	DJI	n.a.	n.a.	No	1,098.0
Egypt	EGY	2005	2014	Yes	108,391.5
Iran	IRN	n.a.	n.a.	No	87,590.2
Morocco	MAR	1992	2003-04	Yes	36,888.7
State of Palestine	PSE	n.a.	n.a.	No	5,076.8
Syria	SYR	n.a.	n.a.	No	21,059.1
Yemen	YEM	1991-92	2013	No	32,641.0
**East Asia and Pacific**					
Cambodia	KHM	2000	2014	Yes	16,497.4
Indonesia	IDN	1997	2017	Yes	272,890.1

Country	ISO3 Alpha-code	Year of Survey Round-*1*	Year of Survey Round-*n*	Included in Analysis	Population
DPR Korea	PRK	n.a.	n.a.	No	25,921.7
Kiribati	KIR	n.a.	n.a.	No	127.7
Lao PDR	LAO	n.a.	n.a.	No	7,373.1
Mongolia	MNG	n.a.	n.a.	No	3,322.2
Myanmar	MMR	n.a.	2015-16	No	53,618.4
Papua New Guinea	PNG	n.a.	2016-18	No	9,850.5
Philippines	PHL	1993	2017	Yes	113,094.3
Samoa	WSM	n.a.	n.a.	No	216.8
Solomon Islands	SLB	n.a.	n.a.	No	699.5
Timor-Leste	TLS	2009-10	2016	Yes	1,310.2
Tunisia	TUN	n.a.	n.a.	No	12,217.2
Vanuatu	VUT	n.a.	n.a.	No	315.4
Vietnam	VNM	1997	2005	Yes	97,093.8
**South Asia**					
Afghanistan	AFG	n.a.	2015	No	39,618.4
Bangladesh	BGD	1993-94	2017-18	Yes	168,414.9
Bhutan	BTN	n.a.	n.a.	No	775.0
India	IND	1992-93	2019-21	Yes	1,402,807.9
Nepal	NPL	1996	2016	Yes	29,698.0
Pakistan	PAK	1990-91	2017-18	Yes	229,280.6
Sri Lanka	LKA	1987	n.a.	No	21,747.0
**Latin America and the Caribbean**					
Belize	BLZ	n.a.	n.a.	No	397.4
Bolivia	BOL	1994	2008	Yes	12,010.0
El Salvador	SLV	n.a.	n.a.	No	6,304.5
Haiti	HTI	1994-95	2016-17	Yes	11,379.1
Honduras	HND	2005-06	2011-12	Yes	10,201.7
Nicaragua	NIC	1998	2001	Yes	6,802.1
**Europe and Central Asia**					
Kyrgyz Rep.	KGZ	1997	2012	Yes	6,477.4
Tajikistan	TJK	2012	2017	Yes	9,643.6
Ukraine	UKR	n.a.	n.a.	No	43,728.6
Uzbekistan	UZB	1996	n.a.	No	33,809.9

Notes: Population estimates were retrieved from World Population Prospects, 2022 version. n.a. = not available.

Figure A- 1: Concentration Curve for Bolivia and the Philippines, in-union women

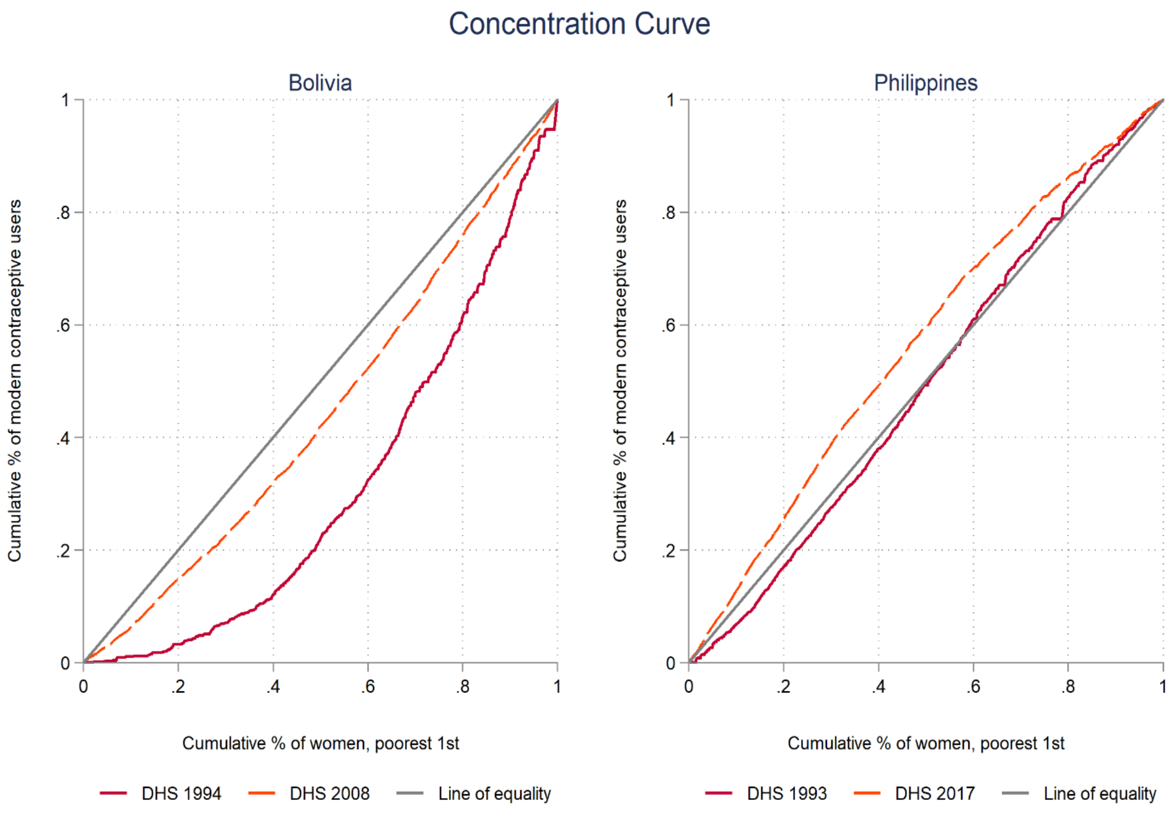

Table A- 2: mCPR and mDFPS reported in the first and most recent DHS round

Country	In-union women					
	Sample		mCRP		mDFPS	
	Round *1*	Round *n*	Round *1*	Round *n*	Round *1*	Round *n*
Benin (1996, 2017-18)	4,264	11,170	3.4	12.4	7.8	25.9
Burkina Faso (1993, 2010)	5,096	13,392	4.2	15.0	8.5	36.9
Burundi (2010, 2016-17)	5,261	9,559	17.7	22.9	32.7	39.4
Cameroon (1991, 2011)	2,737	9,805	4.3	14.4	11.1	30.8
Chad (1996-97, 2014-15)	5,731	13,439	1.2	5.0	5.4	17.6
Comoros (1996, 2012)	1,634	3,291	11.4	14.2	20.1	27.4
Congo (2005, 2011-12)	3,993	6,750	12.7	20.0	19.8	31.7
Congo Dem. Rep. (2007, 2013-14)	6,586	12,448	5.8	7.8	12.2	16.3
Cote d'Ivoire (1994, 2011-12)	5,248	6,453	4.3	12.5	10.3	27.5
Ethiopia (2000, 2016)	9,380	9,824	6.3	35.3	14.2	60.6
Gambia (2013, 2019-20)	6,905	8,083	8.1	17.1	23.8	39.6
Ghana (1998, 2014)	3,694	5,456	18.7	22.2	31.3	39.2
Guinea (1999, 2018)	5,531	7,812	4.2	10.6	13.6	32.1
Kenya (1993, 2014)	4,583	19,036	27.3	53.2	40.1	70.7
Lesotho (2004, 2014)	3,726	3,609	35.2	59.8	51.5	76.1
Liberia (2007, 2019-20)	4,508	4,654	10.3	23.9	21.8	40.9
Madagascar (1997, 2008-09)	4,356	11,903	9.7	29.2	20.7	49.5
Malawi (1992, 2015-16)	3,489	15,952	7.4	58.1	14.9	74.6
Mali (1995-96, 2018)	8,065	8,332	4.5	16.4	13.1	39.9
Mozambique (1997, 2011)	6,260	8,956	5.1	11.3	16.6	31.9
Niger (1998, 2012)	6,118	9,509	4.6	12.2	17.8	40.8
Nigeria (1990, 2018)	6,694	28,888	3.5	12.0	12.9	33.9
Rwanda (1992, 2019-20)	3,698	7,290	12.9	58.4	21.7	75.2
Senegal (1997, 2019)	6,030	5,933	8.1	25.5	16.9	52.6
Sierra Leone (2008, 2019)	5,373	9,837	6.7	20.9	18.4	45.4
South Africa (1998, 2016)	4,948	2,841	55.1	54.0	75.7	77.8
Tanzania (1996, 2015-16)	5,404	8,189	13.3	32.0	30.0	52.9
Togo (1998, 2013-14)	5,976	6,360	7.0	17.3	11.9	32.3
Uganda (1995, 2016)	4,903	11,379	7.8	34.8	17.4	51.6
Zambia (1996, 2013-14)	4,949	9,649	14.4	44.8	28.1	63.8
Zimbabwe (1994, 2015)	3,777	6,015	42.2	65.8	62.7	85.2
Egypt (2005, 2014)	18,134	20,430	56.5	56.9	79.0	80.0
Morocco (1992, 2003-04)	5,118	8,851	35.5	54.8	54.7	73.2
Cambodia (2000, 2014)	9,332	11,668	18.8	38.8	33.0	56.4
Indonesia (1997, 2017)	26,833	34,467	54.7	57.2	77.1	77.1
Philippines (1993, 2017)	9,145	15,445	24.9	40.4	35.4	56.9
Timor-Leste (2009-10, 2016)	7,877	7,628	21.1	24.1	39.2	46.9
Vietnam (1997, 2002)	5,331	5,341	55.8	56.7	66.7	66.6
Bangladesh (1993-94, 2017-18)	8,989	18,895	36.2	51.9	54.6	70.3
India (1992-93, 2019-21)	84,558	512,408	36.3	56.4	59.6	74.1
Nepal (1996, 2016)	7,978	9,904	26.0	42.8	42.8	56.0
Pakistan (1990-91, 2017-18)	6,393	11,902	9.0	25.0	21.3	48.6
Bolivia (1994, 2008)	5,327	10,188	17.8	34.6	24.0	42.8
Haiti (1994-95, 2016-17)	3,076	7,563	13.2	31.8	21.1	44.0
Honduras (2005-06, 2011-12)	11,709	13,178	56.4	63.8	68.7	76.1
Nicaragua (1998, 2001)	8,166	7,678	57.4	66.1	73.4	79.4
Kyrgyz Republic (1997, 2012)	2,677	5,478	48.9	33.7	68.5	62.0
Tajikistan (2012, 2017)	6,388	7,545	25.8	27.1	50.9	52.1

Table A- 3: mCPR and mDFPS in the richest and poorest wealth quintiles in the most recent DHS round, in-union women

Country	mCPR						mDFPS					
	Poorest	Richest	Poorest/ Richest				Poorest	Richest	Poorest/ Richest			
			Coeff.	p-value	Lower bound	Upper bound			Coeff.	p-value	Lower bound	Upper bound
Benin (2017-18)	0.094	0.179	0.524	0.00	0.428	0.643	0.234	0.319	0.736	0.00	0.616	0.879
Burkina Faso (2010)	0.071	0.336	0.212	0.00	0.177	0.254	0.223	0.579	0.384	0.00	0.327	0.452
Burundi (2016-17)	0.222	0.276	0.803	0.00	0.693	0.929	0.394	0.454	0.867	0.03	0.764	0.985
Cameroon (2011)	0.024	0.257	0.093	0.00	0.067	0.130	0.083	0.428	0.194	0.00	0.143	0.262
Chad (2014-15)	0.038	0.106	0.357	0.00	0.248	0.516	0.136	0.277	0.491	0.00	0.350	0.689
Comoros (2012)	0.109	0.142	0.769	0.14	0.540	1.095	0.198	0.297	0.667	0.02	0.477	0.934
Congo (2011-12)	0.095	0.288	0.330	0.00	0.269	0.405	0.160	0.448	0.358	0.00	0.294	0.437
Congo Dem. Rep. (2013-14)	0.033	0.172	0.193	0.00	0.134	0.277	0.080	0.276	0.291	0.00	0.211	0.400
Cote d'Ivoire (2011-12)	0.074	0.201	0.366	0.00	0.275	0.487	0.183	0.426	0.430	0.00	0.332	0.557
Ethiopia (2016)	0.195	0.474	0.410	0.00	0.343	0.491	0.422	0.752	0.561	0.00	0.486	0.648
Gambia (2019-20)	0.157	0.177	0.887	0.30	0.706	1.113	0.385	0.405	0.953	0.57	0.807	1.125
Ghana (2014)	0.212	0.195	1.087	0.48	0.862	1.369	0.398	0.366	1.088	0.36	0.906	1.307
Guinea (2018)	0.044	0.171	0.257	0.00	0.179	0.368	0.161	0.459	0.351	0.00	0.257	0.479
Kenya (2014)	0.292	0.577	0.507	0.00	0.466	0.550	0.456	0.788	0.578	0.00	0.525	0.636
Lesotho (2014)	0.499	0.659	0.757	0.00	0.672	0.852	0.666	0.827	0.806	0.00	0.741	0.877
Liberia (2019-20)	0.235	0.184	1.281	0.12	0.937	1.750	0.409	0.360	1.136	0.37	0.856	1.507
Madagascar (2008-09)	0.176	0.364	0.484	0.00	0.416	0.563	0.410	0.493	0.831	0.00	0.730	0.945
Malawi (2015-16)	0.532	0.606	0.878	0.00	0.829	0.930	0.710	0.772	0.921	0.00	0.883	0.961
Mali (2018)	0.110	0.235	0.468	0.00	0.359	0.611	0.265	0.542	0.489	0.00	0.389	0.615
Mozambique (2011)	0.029	0.295	0.098	0.00	0.068	0.141	0.112	0.512	0.219	0.00	0.154	0.312
Niger (2012)	0.087	0.237	0.367	0.00	0.284	0.475	0.315	0.567	0.556	0.00	0.452	0.683
Nigeria (2018)	0.037	0.223	0.165	0.00	0.137	0.199	0.178	0.432	0.412	0.00	0.352	0.481
Rwanda (2019-20)	0.602	0.518	1.161	0.00	1.080	1.248	0.757	0.715	1.059	0.05	1.000	1.121
Senegal (2019)	0.166	0.326	0.510	0.00	0.404	0.645	0.406	0.628	0.646	0.00	0.530	0.787
Sierra Leone (2019)	0.158	0.258	0.613	0.00	0.518	0.726	0.397	0.503	0.789	0.00	0.681	0.914
South Africa (2016)	0.536	0.551	0.974	0.70	0.851	1.114	0.767	0.787	0.975	0.61	0.881	1.078
Tanzania (2015-16)	0.203	0.354	0.574	0.00	0.488	0.676	0.392	0.538	0.729	0.00	0.637	0.835
Togo (2013-14)	0.155	0.208	0.745	0.00	0.607	0.914	0.300	0.380	0.788	0.01	0.655	0.949
Uganda (2016)	0.224	0.421	0.532	0.00	0.475	0.597	0.363	0.600	0.605	0.00	0.547	0.669
Zambia (2013-14)	0.313	0.583	0.537	0.00	0.482	0.598	0.491	0.784	0.626	0.00	0.574	0.683
Zimbabwe (2015)	0.618	0.723	0.856	0.00	0.799	0.916	0.804	0.903	0.891	0.00	0.852	0.931
Egypt (2014)	0.542	0.593	0.915	0.00	0.864	0.969	0.761	0.819	0.929	0.00	0.896	0.963
Morocco (2003-04)	0.514	0.568	0.905	0.01	0.842	0.973	0.722	0.719	1.004	0.89	0.949	1.063
Cambodia (2014)	0.396	0.346	1.143	0.01	1.036	1.262	0.567	0.482	1.178	0.00	1.085	1.278
Indonesia (2017)	0.563	0.523	1.078	0.00	1.033	1.124	0.785	0.708	1.109	0.00	1.077	1.142
Philippines (2017)	0.438	0.334	1.312	0.00	1.170	1.472	0.599	0.512	1.169	0.00	1.062	1.286
Timor-Leste (2016)	0.234	0.255	0.917	0.34	0.767	1.096	0.458	0.470	0.973	0.71	0.846	1.120
Vietnam (2002)	0.579	0.516	1.120	0.04	1.007	1.247	0.693	0.625	1.110	0.04	1.004	1.227
Bangladesh (2017-18)	0.572	0.488	1.171	0.00	1.106	1.240	0.751	0.679	1.106	0.00	1.060	1.155
India (2019-21)	0.507	0.587	0.863	0.00	0.851	0.875	0.689	0.753	0.915	0.00	0.905	0.924
Nepal (2016)	0.418	0.430	0.972	0.60	0.876	1.080	0.550	0.532	1.032	0.53	0.934	1.141
Pakistan (2017-18)	0.171	0.297	0.576	0.00	0.474	0.699	0.401	0.511	0.785	0.00	0.683	0.902

Country	mCPR						mDFPS					
	Poorest	Richest	Poorest/ Richest				Poorest	Richest	Poorest/ Richest			
			Coeff.	p-value	Lower bound	Upper bound			Coeff.	p-value	Lower bound	Upper bound
Bolivia (2008)	0.226	0.465	0.487	0.00	0.430	0.551	0.282	0.580	0.486	0.00	0.433	0.545
Haiti (2016-17)	0.283	0.283	0.999	0.99	0.848	1.176	0.378	0.454	0.833	0.01	0.727	0.955
Honduras (2011-12)	0.551	0.674	0.818	0.00	0.776	0.862	0.680	0.790	0.861	0.00	0.825	0.899
Nicaragua (2001)	0.502	0.710	0.708	0.00	0.664	0.754	0.646	0.845	0.765	0.00	0.727	0.805
Kyrgyz Republic (2012)	0.364	0.340	1.069	0.36	0.926	1.233	0.680	0.627	1.085	0.13	0.976	1.205
Tajikistan (2017)	0.255	0.308	0.827	0.02	0.702	0.974	0.494	0.571	0.864	0.02	0.765	0.977

Notes: Poorest/ richest ratios are not adjusted for demographic characteristics.

Table A- 4: Concentration indices (CI) for mCPR and mDFPS in the first and most recent DHS, in-union women

Country	mCRP		mDFPS	
	Round *1*	Round *n*	Round *1*	Round *n*
**Total**	**0.152**	**0.079**	**0.095**	**0.038**
Benin (1996, 2017-18)	0.426	0.140	0.332	0.076
Burkina Faso (1993, 2010)	0.700	0.336	0.647	0.218
Burundi (2010, 2016-17)	0.120	0.029	0.091	0.017
Cameroon (1991, 2011)	0.537	0.348	0.377	0.210
Chad (1996-97, 2014-15)	0.708	0.189	0.593	0.139
Comoros (1996, 2012)	0.156	0.062	0.184	0.085
Congo (2005, 2011-12)	0.186	0.186	0.182	0.173
Congo Dem. Rep. (2007, 2013-14)	0.387	0.344	0.298	0.261
Cote d'Ivoire (1994, 2011-12)	0.488	0.220	0.383	0.189
Ethiopia (2000, 2016)	0.506	0.153	0.448	0.102
Gambia (2013, 2019-20)	0.276	0.045	0.212	0.034
Ghana (1998, 2014)	0.179	-0.022	0.167	-0.021
Guinea (1999, 2018)	0.436	0.245	0.325	0.179
Kenya (1993, 2014)	0.285	0.089	0.234	0.079
Lesotho (2004, 2014)	0.222	0.049	0.185	0.042
Liberia (2007, 2019-20)	0.284	-0.023	0.219	-0.012
Madagascar (1997, 2008-09)	0.461	0.139	0.340	0.023
Malawi (1992, 2015-16)	0.334	0.021	0.287	0.014
Mali (1995-96, 2018)	0.597	0.172	0.482	0.151
Mozambique (1997, 2011)	0.557	0.443	0.437	0.279
Niger (1998, 2012)	0.599	0.246	0.467	0.141
Nigeria (1990, 2018)	0.561	0.337	0.436	0.148
Rwanda (1992, 2019-20)	0.107	-0.024	0.115	-0.008
Senegal (1997, 2019)	0.550	0.142	0.483	0.089
Sierra Leone (2008, 2019)	0.420	0.113	0.340	0.059
South Africa (1998, 2016)	0.139	0.013	0.098	0.012
Tanzania (1996, 2015-16)	0.355	0.107	0.276	0.056
Togo (1998, 2013-14)	0.257	0.059	0.255	0.044
Uganda (1995, 2016)	0.533	0.115	0.437	0.093
Zambia (1996, 2013-14)	0.354	0.117	0.267	0.089
Zimbabwe (1994, 2015)	0.134	0.037	0.104	0.022
Egypt (2005, 2014)	0.036	0.020	0.022	0.017
Morocco (1992, 2003-04)	0.172	0.017	0.111	-0.004
Cambodia (2000, 2014)	0.143	-0.030	0.132	-0.038
Indonesia (1997, 2017)	0.037	-0.020	0.013	-0.021
Philippines (1993, 2017)	0.128	-0.057	0.128	-0.030
Timor-Leste (2009-10, 2016)	0.174	0.027	0.124	0.008
Vietnam (1997, 2002)	0.026	-0.019	0.001	-0.020
Bangladesh (1993-94, 2017-18)	0.038	-0.028	0.006	-0.017
India (1992-93, 2019-21)	0.152	0.028	0.082	0.017
Nepal (1996, 2016)	0.204	-0.001	0.152	-0.008
Pakistan (1990-91, 2017-18)	0.462	0.101	0.330	0.044
Bolivia (1994, 2008)	0.445	0.147	0.413	0.146
Haiti (1994-95, 2016-17)	0.284	-0.006	0.236	0.030
Honduras (2005-06, 2011-12)	0.080	0.038	0.061	0.029
Nicaragua (1998, 2001)	0.085	0.058	0.064	0.044
Kyrgyz Republic (1997, 2012)	0.047	-0.023	0.012	-0.027
Tajikistan (2012, 2017)	0.076	0.046	0.052	0.032

Table A- 5: Adjusted average annual rate of change in mCPR between the first and most recent DHS round, in-union women, by wealth quintile

Country	Total			Poorest			Poorer			Middle			Richer			Richest		
	Rate	Lower bound	Upper bound	Rate	Lower bound	Upper bound	Rate	Lower bound	Upper bound	Rate	Lower bound	Upper bound	Rate	Lower bound	Upper bound	Rate	Lower bound	Upper bound
**Total**	**2.1**	**2.1**	**2.2**	**3.1**	**3.0**	**3.2**	**2.8**	**2.7**	**2.9**	**2.3**	**2.2**	**2.4**	**1.9**	**1.8**	**2.0**	**1.3**	**1.3**	**1.4**
Benin (1996, 2017-18)	6.3	5.3	7.3	9.3	6.7	11.8	9.9	6.9	12.9	9.8	7.0	12.7	5.4	3.6	7.1	3.8	2.6	5.0
Burkina Faso (1993, 2010)	7.2	6.3	8.1	13.0	8.4	17.6	19.1	12.5	25.8	13.3	8.9	17.7	11.1	7.7	14.5	4.1	3.3	5.0
Burundi (2010, 2016-17)	4.5	2.7	6.4	7.4	3.9	11.0	8.0	4.5	11.5	4.6	1.3	7.9	2.8	-0.6	6.2	1.2	-1.3	3.8
Cameroon (1991, 2011)	5.7	4.6	6.8	5.7	-0.3	11.8	9.1	5.1	13.0	8.3	5.0	11.6	8.3	6.2	10.4	4.2	3.2	5.2
Chad (1996-97, 2014-15)	8.0	6.5	9.5	23.0	11.9	34.2	15.8	9.2	22.3	18.8	7.7	29.8	6.6	2.2	10.9	4.0	2.5	5.6
Comoros (1996, 2012)	1.7	0.4	2.9	3.4	0.2	6.6	1.2	-1.1	3.5	2.2	-0.3	4.7	3.9	1.5	6.2	-0.1	-1.9	1.7
Congo (2005, 2011-12)	6.5	4.3	8.7	1.6	-3.3	6.5	11.8	5.4	18.3	9.1	4.8	13.5	5.6	1.6	9.6	7.8	3.8	11.8
Congo Dem. Rep. (2007, 2013-14)	6.6	2.6	10.6	3.3	-8.2	14.8	10.6	-0.1	21.3	4.4	-4.9	13.6	9.0	1.5	16.5	2.4	-1.4	6.2
Cote d'Ivoire (1994, 2011-12)	6.3	5.3	7.3	11.2	8.0	14.4	9.6	6.9	12.3	10.4	7.8	13.0	6.4	4.7	8.2	3.7	2.2	5.2
Ethiopia (2000, 2016)	10.5	9.5	11.5	12.1	9.6	14.6	15.1	12.5	17.8	18.5	15.6	21.3	15.2	12.8	17.6	4.8	3.7	5.9
Gambia (2013, 2019-20)	11.0	8.0	14.0	21.2	15.7	26.8	19.9	13.9	25.9	17.5	11.0	24.0	10.7	5.9	15.4	2.7	-1.9	7.4
Ghana (1998, 2014)	1.9	0.8	3.0	8.2	5.8	10.7	2.8	0.5	5.0	3.0	0.9	5.2	1.4	-0.5	3.2	-1.8	-3.7	0.0
Guinea (1999, 2018)	5.1	3.9	6.2	8.0	4.4	11.7	8.9	5.9	11.9	6.8	4.5	9.2	4.0	2.1	5.9	3.6	2.0	5.2
Kenya (1993, 2014)	3.0	2.6	3.5	4.8	3.7	5.8	6.0	5.2	6.8	3.8	3.2	4.4	3.0	2.3	3.6	1.8	1.3	2.3
Lesotho (2004, 2014)	5.1	4.4	5.9	12.0	9.9	14.1	8.7	7.2	10.3	6.1	4.8	7.4	4.7	3.5	5.9	2.5	1.3	3.7
Liberia (2007, 2019-20)	6.9	5.5	8.3	16.0	11.7	20.3	12.9	9.6	16.2	5.1	2.4	7.7	6.3	4.5	8.1	1.3	-2.0	4.5
Madagascar (1997, 2008-09)	10.6	9.0	12.3	19.6	13.6	25.6	19.4	13.5	25.3	13.2	9.5	16.9	10.4	7.8	13.0	4.8	3.6	5.9
Malawi (1992, 2015-16)	9.3	8.6	9.9	11.7	9.9	13.5	12.3	10.4	14.2	10.5	9.1	12.0	9.5	8.3	10.7	6.0	5.3	6.7
Mali (1995-96, 2018)	6.0	5.3	6.8	13.8	11.2	16.5	8.7	6.3	11.1	9.7	7.9	11.5	7.1	6.0	8.3	2.3	1.4	3.1
Mozambique (1997, 2011)	4.7	3.1	6.4	7.9	2.2	13.6	7.6	3.4	11.7	5.5	2.1	8.9	7.0	4.3	9.7	4.0	2.3	5.6
Niger (1998, 2012)	7.2	5.9	8.5	17.4	10.5	24.3	11.0	6.6	15.4	9.5	6.0	13.0	10.4	7.6	13.2	2.6	1.3	3.9
Nigeria (1990, 2018)	3.6	2.9	4.3	7.0	4.3	9.7	4.6	2.6	6.7	6.4	4.5	8.3	5.8	4.4	7.2	2.9	2.3	3.4
Rwanda (1992, 2019-20)	6.2	5.7	6.7	6.5	5.7	7.4	6.9	5.9	8.0	6.6	5.7	7.4	6.1	5.3	7.0	4.9	4.1	5.6
Senegal (1997, 2019)	5.5	4.6	6.4	13.2	10.9	15.5	12.1	10.0	14.2	8.7	7.2	10.1	5.7	4.6	6.9	2.0	1.0	2.9
Sierra Leone (2008, 2019)	10.2	8.7	11.7	15.4	11.5	19.2	17.3	13.5	21.2	17.0	13.4	20.7	9.7	7.4	11.9	3.9	1.7	6.1
South Africa (1998, 2016)	-0.1	-0.4	0.2	2.7	2.0	3.4	1.1	0.4	1.8	0.4	-0.3	1.0	0.1	-0.5	0.6	-1.1	-1.6	-0.5
Tanzania (1996, 2015-16)	4.5	3.9	5.2	7.5	5.9	9.1	7.2	5.8	8.7	6.8	5.7	8.0	5.9	5.0	6.8	1.5	0.8	2.3
Togo (1998, 2013-14)	6.2	5.2	7.2	10.2	7.9	12.5	8.5	6.2	10.9	6.2	4.4	8.0	5.9	4.1	7.7	4.2	2.6	5.7
Uganda (1995, 2016)	6.9	6.2	7.7	11.3	9.3	13.4	11.8	10.0	13.6	10.3	8.8	11.8	9.0	7.6	10.4	2.6	1.9	3.3
Zambia (1996, 2013-14)	6.9	6.3	7.5	10.4	8.9	11.9	7.8	6.6	9.0	9.9	8.5	11.3	6.4	5.6	7.2	4.2	3.3	5.0
Zimbabwe (1994, 2015)	2.3	2.0	2.6	3.3	2.7	4.0	3.3	2.6	4.0	2.7	2.1	3.2	1.9	1.5	2.2	1.6	1.2	2.0
Egypt (2005, 2014)	0.6	0.4	0.9	1.0	0.3	1.7	0.4	-0.1	1.0	1.1	0.6	1.6	0.1	-0.3	0.6	0.1	-0.4	0.6
Morocco (1992, 2003-04)	4.5	3.8	5.2	10.3	7.9	12.7	6.2	4.5	7.9	4.3	3.4	5.3	3.4	2.4	4.3	2.5	1.8	3.1
Cambodia (2000, 2014)	6.2	5.6	6.7	9.0	7.8	10.2	8.4	7.3	9.4	6.0	5.0	6.9	6.3	5.2	7.4	3.4	2.5	4.4
Indonesia (1997, 2017)	0.7	0.6	0.8	1.2	0.9	1.6	1.0	0.7	1.2	0.9	0.6	1.1	0.6	0.3	0.9	0.3	0.1	0.5
Philippines (1993, 2017)	2.5	2.3	2.7	4.5	4.0	5.0	3.4	3.0	3.8	2.5	2.1	2.9	1.7	1.3	2.2	1.4	0.8	1.9
Timor-Leste (2009-10, 2016)	3.1	1.8	4.4	8.1	5.4	10.7	6.4	3.8	9.0	6.8	4.2	9.4	2.9	0.6	5.2	-2.1	-4.5	0.3
Vietnam (1997, 2002)	0.7	-0.5	2.0	4.5	1.8	7.2	0.4	-2.0	2.7	-0.2	-2.0	1.7	0.2	-1.6	2.0	-0.5	-2.7	1.7
Bangladesh (1993-94, 2014)	1.9	1.7	2.1	2.4	2.0	2.9	2.1	1.7	2.4	2.0	1.6	2.3	2.0	1.6	2.4	0.9	0.6	1.3
India (1992-93, 2019-21)	1.7	1.6	1.7	2.6	2.4	2.8	2.6	2.4	2.8	1.9	1.8	2.1	1.5	1.4	1.7	0.9	0.8	1.0
Nepal (1996, 2016)	2.1	1.6	2.5	4.7	3.8	5.6	3.6	2.8	4.3	2.9	2.1	3.7	2.3	1.5	3.1	-0.3	-0.8	0.2
Pakistan (1990-91, 2017-18)	4.3	3.9	4.7	9.9	7.1	12.6	6.5	5.2	7.9	6.2	4.8	7.6	4.8	4.0	5.6	2.1	1.5	2.7
Bolivia (1994, 2008)	4.8	4.1	5.4	19.0	15.0	23.0	10.3	8.2	12.4	6.2	5.1	7.4	4.8	3.9	5.7	1.3	0.6	1.9
Haiti (1994-95, 2016-17)	4.3	3.7	4.9	8.0	5.8	10.2	7.1	5.5	8.7	5.1	3.9	6.2	2.7	1.7	3.6	2.3	1.3	3.3
Honduras (2005-06, 2011-12)	2.3	1.8	2.7	4.8	3.7	5.9	3.2	2.3	4.0	2.2	1.4	3.1	1.8	1.0	2.5	1.3	0.5	2.2
Nicaragua (1998, 2001)	4.9	3.8	6.0	7.5	4.4	10.6	6.0	3.9	8.2	5.7	4.0	7.4	3.0	1.2	4.8	3.5	1.6	5.5
Kyrgyz Republic (1997, 2012)	-2.0	-2.6	-1.4	-0.3	-1.5	0.8	-1.1	-2.4	0.2	-1.8	-3.1	-0.4	-3.2	-4.2	-2.2	-3.2	-4.2	-2.3
Tajikistan (2012, 2017)	2.4	0.7	4.2	4.0	0.0	8.1	2.8	-0.8	6.3	3.5	-0.2	7.3	3.7	0.5	6.9	-1.0	-3.3	1.4

Table A- 6: Adjusted average annual rate of change in mDFPS between the first and most recent DHS round, in-union women, by wealth quintile

Country	Total			Poorest			Poorer			Middle			Richer			Richest		
	Rate	Lower bound	Upper bound	Rate	Lower bound	Upper bound	Rate	Lower bound	Upper bound	Rate	Lower bound	Upper bound	Rate	Lower bound	Upper bound	Rate	Lower bound	Upper bound
**Total**	**1.0**	**0.9**	**1.0**	**1.6**	**1.5**	**1.7**	**1.3**	**1.2**	**1.4**	**1.0**	**0.9**	**1.1**	**0.8**	**0.8**	**0.9**	**0.6**	**0.5**	**0.6**
Benin (1996, 2017-18)	5.7	4.8	6.6	8.5	6.1	10.8	8.8	6.2	11.5	9.0	6.2	11.7	4.8	3.3	6.4	3.4	2.3	4.6
Burkina Faso (1993, 2010)	8.0	7.1	8.9	15.5	10.9	20.1	20.4	13.8	27.0	14.0	9.8	18.2	11.8	8.5	15.2	4.0	3.4	4.7
Burundi (2010, 2016-17)	3.2	1.7	4.7	5.8	2.7	8.8	6.8	3.6	10.0	2.6	-0.2	5.5	0.9	-2.0	3.9	1.0	-1.1	3.2
Cameroon (1991, 2011)	4.5	3.6	5.3	4.7	-0.3	9.7	7.3	3.9	10.8	6.8	3.9	9.6	6.3	4.5	8.0	3.3	2.4	4.3
Chad (1996-97, 2014-15)	6.8	5.5	8.2	21.8	10.7	33.0	13.8	7.5	20.1	17.2	15.7	18.7	6.3	2.5	10.1	3.4	2.0	4.7
Comoros (1996, 2012)	1.7	0.5	2.8	3.7	0.7	6.6	1.7	-0.3	3.7	1.9	-0.2	4.1	3.4	1.1	5.8	-0.2	-2.0	1.5
Congo (2005, 2011-12)	7.0	5.0	8.9	2.4	-2.3	7.1	11.0	5.4	16.5	10.2	6.3	14.1	5.7	1.8	9.5	7.5	4.1	10.9
Congo Dem. Rep. (2007, 2013-14)	5.8	2.3	9.4	3.1	-8.1	14.4	9.7	-0.5	19.8	4.0	-4.3	12.3	7.5	0.7	14.2	3.5	0.2	6.9
Cote d'Ivoire (1994, 2011-12)	5.3	4.4	6.2	8.9	5.8	12.0	8.1	5.3	10.9	8.8	6.2	11.3	5.5	3.8	7.3	3.7	2.4	5.0
Ethiopia (2000, 2016)	8.7	7.9	9.6	11.2	9.0	13.4	12.6	10.2	14.9	15.3	13.1	17.6	12.5	10.3	14.8	4.0	3.1	4.9
Gambia (2013, 2019-20)	7.3	5.0	9.7	15.8	10.9	20.7	14.6	9.3	19.9	11.5	5.9	17.1	6.7	2.7	10.8	0.5	-2.9	3.8
Ghana (1998, 2014)	1.9	1.0	2.9	8.6	6.5	10.8	2.5	0.7	4.4	2.5	0.5	4.4	0.9	-0.7	2.4	-1.6	-3.2	-0.1
Guinea (1999, 2018)	4.2	3.2	5.2	6.7	3.4	10.1	7.4	4.7	10.0	5.5	3.5	7.5	3.0	1.5	4.6	3.3	2.0	4.5
Kenya (1993, 2014)	2.2	1.9	2.5	4.4	3.4	5.4	4.3	3.5	5.1	2.7	2.2	3.2	2.1	1.5	2.7	1.3	0.8	1.7
Lesotho (2004, 2014)	3.4	2.8	3.9	9.3	7.4	11.1	6.1	4.8	7.4	4.0	3.0	5.1	3.0	2.0	4.0	1.2	0.3	2.0
Liberia (2007, 2019-20)	5.0	3.8	6.3	12.4	8.4	16.3	9.9	7.0	12.8	3.9	1.7	6.2	4.4	2.7	6.1	0.8	-1.9	3.5
Madagascar (1997, 2008-09)	7.8	6.4	9.2	18.3	12.7	24.0	14.8	9.4	20.2	9.9	6.7	13.2	7.7	5.3	10.0	3.1	2.1	4.2
Malawi (1992, 2015-16)	7.1	6.5	7.7	9.6	8.0	11.2	9.8	7.9	11.6	7.9	6.5	9.2	7.1	6.0	8.3	4.5	3.8	5.2
Mali (1995-96, 2018)	4.9	4.3	5.5	11.7	9.3	14.1	7.5	5.8	9.3	8.3	6.8	9.8	6.0	5.1	6.9	2.1	1.4	2.8
Mozambique (1997, 2011)	3.8	2.4	5.2	8.3	5.0	11.7	6.5	4.0	9.0	4.0	1.3	6.7	5.7	3.3	8.1	2.0	0.6	3.3
Niger (1998, 2012)	6.1	5.0	7.3	15.8	9.2	22.5	10.1	6.3	14.0	8.0	5.0	11.1	9.4	7.0	11.9	2.1	1.2	3.1
Nigeria (1990, 2018)	2.9	2.4	3.5	7.2	4.9	9.6	4.5	2.6	6.3	5.1	3.5	6.7	3.9	2.6	5.2	1.6	1.1	2.1
Rwanda (1992, 2019-20)	4.6	4.1	5.0	5.3	4.6	6.1	4.9	4.0	5.8	5.0	4.2	5.7	4.2	3.4	5.1	3.3	2.7	3.9
Senegal (1997, 2019)	4.9	4.1	5.6	12.7	10.5	15.0	10.8	8.8	12.7	7.7	6.5	9.0	4.5	3.4	5.6	1.7	0.9	2.5
Sierra Leone (2008, 2019)	7.8	6.5	9.1	13.0	9.5	16.5	13.9	10.5	17.4	13.5	10.1	16.9	7.3	5.3	9.3	2.2	0.4	4.0
South Africa (1998, 2016)	-0.1	-0.3	0.2	1.5	0.9	2.0	0.6	0.1	1.2	0.3	-0.1	0.7	-0.1	-0.5	0.3	-0.7	-1.1	-0.3
Tanzania (1996, 2015-16)	2.6	2.1	3.2	5.9	4.4	7.3	4.9	3.7	6.0	4.4	3.5	5.3	3.6	2.8	4.4	0.1	-0.5	0.7
Togo (1998, 2013-14)	6.3	5.4	7.3	11.0	8.8	13.1	8.3	6.3	10.2	6.5	4.7	8.2	5.3	3.6	7.0	3.8	2.4	5.2
Uganda (1995, 2016)	4.9	4.3	5.5	8.7	6.8	10.6	9.2	7.5	11.0	7.9	6.7	9.0	6.5	5.3	7.8	1.8	1.3	2.4
Zambia (1996, 2013-14)	4.7	4.2	5.2	8.1	6.8	9.5	5.3	4.3	6.3	7.1	5.8	8.4	4.3	3.8	4.9	2.6	1.9	3.2
Zimbabwe (1994, 2015)	1.3	1.1	1.4	2.2	1.7	2.7	1.9	1.4	2.3	1.8	1.4	2.1	0.8	0.5	1.1	0.7	0.4	1.0
Egypt (2005, 2014)	0.1	-0.1	0.3	0.0	-0.5	0.5	-0.3	-0.7	0.1	0.3	-0.1	0.6	-0.1	-0.4	0.2	0.1	-0.3	0.4
Morocco (1992, 2003-04)	2.6	2.0	3.1	7.2	5.2	9.2	3.5	2.1	4.8	2.2	1.4	3.0	1.5	0.8	2.3	1.1	0.5	1.7
Cambodia (2000, 2014)	4.0	3.5	4.5	6.9	5.8	8.0	5.9	4.9	6.8	3.6	2.6	4.6	3.7	2.9	4.6	1.4	0.5	2.2
Indonesia (1997, 2017)	0.1	0.0	0.1	0.4	0.2	0.6	0.2	0.0	0.3	0.0	-0.2	0.2	0.0	-0.2	0.2	-0.3	-0.4	-0.1
Philippines (1993, 2017)	2.0	1.8	2.2	3.9	3.4	4.4	2.7	2.3	3.1	2.0	1.6	2.3	1.2	0.8	1.6	0.8	0.3	1.3
Timor-Leste (2009-10, 2016)	2.8	1.7	3.9	7.2	4.9	9.4	4.9	2.7	7.2	6.5	4.4	8.7	1.6	-0.4	3.6	-1.1	-3.0	0.9
Vietnam (1997, 2002)	0.2	-0.9	1.4	2.5	0.3	4.7	-0.1	-2.2	2.0	-1.1	-2.8	0.5	-0.1	-1.6	1.5	0.2	-1.8	2.2
Bangladesh (1993-94, 2014)	1.0	0.8	1.1	1.0	0.7	1.3	1.0	0.7	1.3	1.0	0.7	1.3	1.1	0.8	1.5	0.5	0.3	0.8
India (1992-93, 2019-21)	0.6	0.5	0.7	1.1	1.0	1.3	1.2	1.0	1.3	0.7	0.6	0.8	0.5	0.4	0.6	0.3	0.2	0.4
Nepal (1996, 2016)	0.7	0.3	1.0	2.7	1.9	3.4	1.6	0.9	2.3	1.3	0.8	1.9	0.8	0.2	1.5	-1.2	-1.6	-0.8
Pakistan (1990-91, 2017-18)	3.0	2.6	3.4	8.2	5.8	10.6	5.0	3.8	6.3	4.2	3.3	5.1	3.3	2.7	3.9	1.3	0.8	1.8
Bolivia (1994, 2008)	3.9	3.3	4.5	17.6	15.0	20.1	9.1	7.0	11.1	5.4	4.3	6.5	4.1	3.2	4.9	0.8	0.2	1.5
Haiti (1994-95, 2016-17)	3.2	2.6	3.8	6.1	4.1	8.1	5.7	4.1	7.4	3.8	2.7	4.9	1.6	0.8	2.4	1.9	1.0	2.7
Honduras (2005-06, 2011-12)	1.5	1.2	1.9	3.6	2.7	4.5	2.1	1.4	2.8	1.1	0.4	1.8	1.2	0.6	1.8	0.9	0.2	1.6
Nicaragua (1998, 2001)	2.5	1.7	3.3	4.1	1.6	6.7	2.8	1.2	4.4	3.5	2.2	4.8	0.9	-0.4	2.3	1.8	0.3	3.3
Kyrgyz Republic (1997, 2012)	-0.7	-1.1	-0.3	0.3	-0.6	1.1	-0.4	-1.4	0.5	-1.2	-2.1	-0.4	-1.5	-2.2	-0.8	-0.7	-1.4	0.0
Tajikistan (2012, 2017)	0.7	-0.7	2.0	1.5	-1.7	4.7	0.5	-2.5	3.4	0.3	-2.6	3.3	2.5	-0.1	5.1	-1.0	-2.8	0.7

Table A- 7: Average annual rate of change in mCPR and mDFPS between the first and most recent DHS collected during the COVID-19 pandemic by wealth quintiles, and concentration indices, in-union women

Indicator and country	Survey years	Total	Poorest	Poorer	Middle	Richer	Richest	Concentration Index
		Rate (95% CI)	Rate (95% CI)	Rate (95% CI)	Rate (95% CI)	Rate (95% CI)	Rate (95% CI)	Round *n*
mCPR								
Burkina Faso	Pre-COVID-19 (1993, 2010)	7.4 (6.3, 8.4)	13.0 (8.4, 17.6)	19.2 (12.6, 25.9)	13.5 (9.1, 17.8)	11.3 (7.9, 14.7)	4.2 (3.3, 5.0)	0.336
	COVID-19 (1993, 2021)	7.4 (6.8, 8.0)	12.6 (9.8, 15.3)	16.2 (12.2, 20.2)	12.8 (10.1, 15.4)	9.8 (7.8, 11.9)	3.5 (2.9, 4.0)	0.088
Cambodia	Pre-COVID-19 (2000, 2014)	6.2 (5.6, 6.7)	8.9 (7.7, 10.2)	8.4 (7.4, 9.4)	6.0 (5.0, 6.9)	6.3 (5.2, 7.4)	3.4 (2.5, 4.4)	-0.030
	COVID-19 (2000, 2021-22)	5.2 (4.9, 5.6)	7.5 (6.7, 8.2)	6.3 (5.6, 7.0)	4.9 (4.2, 5.5)	5.1 (4.3, 5.9)	3.8 (3.1, 4.5)	-0.030
Cote d'Ivoire	Pre-COVID-19 (1994, 2011-12)	6.4 (5.3, 7.4)	11.3 (8.1, 14.5)	9.1 (6.5, 11.7)	10.3 (7.7, 12.8)	6.5 (4.8, 8.3)	3.7 (2.2, 5.3)	0.220
	COVID-19 (1994, 2021)	5.6 (5.0, 6.2)	8.8 (6.9, 10.6)	7.2 (5.6, 8.7)	8.8 (7.3, 10.4)	6.4 (5.2, 7.6)	3.3 (2.3, 4.2)	0.159
Kenya	Pre-COVID-19 (1993, 2014)	3.2 (2.8, 3.6)	5.1 (4.1, 6.1)	6.0 (5.2, 6.8)	3.8 (3.2, 4.3)	2.8 (2.1, 3.4)	1.8 (1.3, 2.2)	0.089
	COVID-19 (1993, 2022)	2.6 (2.3, 2.9)	4.9 (4.2, 5.6)	4.9 (4.3, 5.4)	3.0 (2.6, 3.4)	2.0 (1.5, 2.5)	1.3 (1.0, 1.7)	0.034
Madagascar	Pre-COVID-19 (1997, 2008-09)	10.1 (8.5,11.8)	19.3 (13.7,24.9)	19.0 (13.3,24.6)	13.1 (9.4,16.7)	10.2 (7.7,12.8)	4.9 (3.7,6.1)	0.139
	COVID-19 (1997, 2021)	6.2 (5.5, 6.9)	10.7 (8.1, 13.3)	11.2 (8.7, 13.8)	8.0 (6.3, 9.6)	5.9 (4.7, 7.1)	3.0 (2.4, 3.5)	0.038
Nepal	Pre-COVID-19 (1996, 2016)	2.5 (2.1, 2.9)	5.1 (4.2, 6.0)	3.8 (3.1, 4.5)	3.2 (2.5, 3.9)	2.3 (1.6, 2.9)	0.1 (-0.4, 0.6)	-0.001
	COVID-19 (1996, 2022)	2.1 (1.8, 2.4)	4.3 (3.7, 4.9)	3.2 (2.7, 3.7)	2.7 (2.2, 3.3)	1.8 (1.2, 2.3)	-0.1 (-0.6, 0.4)	-0.039
Philippines	Pre-COVID-19 (1993, 2017)	2.5 (2.2, 2.7)	4.5 (4.0, 5.0)	3.4 (3.0, 3.8)	2.5 (2.1, 2.9)	1.7 (1.3, 2.2)	1.3 (0.8, 1.9)	-0.057
	COVID-19 (1993, 2022)	2.3 (2.1, 2.5)	4.0 (3.6, 4.4)	3.0 (2.6, 3.3)	2.4 (2.1, 2.8)	1.3 (0.9, 1.8)	1.9 (1.5, 2.3)	-0.048
**COVID Total**		**3.1 (2.9, 3.2)**	**4.3 (4.0, 4.7)**	**4.2 (3.9, 4.5)**	**3.4 (3.2, 3.6)**	**2.9 (2.7, 3.2)**	**2.0 (1.8, 2.2)**	0.349
mDFPS								
Burkina Faso	Pre-COVID-19 (1993, 2010)	8.1 (7.1, 9.1)	15.5 (10.9, 20.1)	20.4 (13.9, 27.0)	14.1 (9.9, 18.3)	12 (8.6, 15.3)	4.0 (3.4, 4.7)	0.218
	COVID-19 (1993, 2021)	7.0 (6.4, 7.6)	12.9 (10.2, 15.7)	15.5 (11.5, 19.5)	11.7 (9.1, 14.3)	9.2 (7.2, 11.2)	3.2 (2.8, 3.6)	0.033
Cambodia	Pre-COVID-19 (2000, 2014)	4.0 (3.5, 4.5)	6.9 (5.8, 8.0)	5.9 (5.0, 6.8)	3.6 (2.6, 4.6)	3.7 (2.9, 4.6)	1.3 (0.5, 2.2)	-0.038
	COVID-19 (2000, 2021-22)	3.0 (2.6, 3.3)	5.4 (4.7, 6.2)	3.7 (3.0, 4.3)	2.5 (1.9, 3.1)	2.6 (2.0, 3.2)	1.6 (1.0, 2.2)	-0.035
Cote d'Ivoire	Pre-COVID-19 (1994, 2011-12)	5.2 (4.2, 6.1)	9.0 (5.9, 12.1)	7.5 (4.8, 10.1)	8.7 (6.2, 11.2)	5.5 (3.8, 7.3)	3.7 (2.5, 5.0)	0.189
	COVID-19 (1994, 2021)	4.8 (4.3, 5.4)	7.5 (5.7, 9.3)	6.2 (4.6, 7.8)	7.7 (6.2, 9.2)	5.6 (4.5, 6.7)	3.0 (2.2, 3.8)	0.114
Kenya	Pre-COVID-19 (1993, 2014)	2.3 (2.0, 2.7)	4.6 (3.6, 5.5)	4.3 (3.6, 5.1)	2.6 (2.1, 3.1)	2.1 (1.5, 2.7)	1.3 (0.9, 1.7)	0.079
	COVID-19 (1993, 2022)	2.0 (1.7, 2.2)	4.3 (3.7, 5.0)	3.7 (3.2, 4.3)	2.2 (1.8, 2.6)	1.5 (1.1, 1.9)	0.9 (0.6, 1.2)	0.020
Madagascar	Pre-COVID-19 (1997, 2008-09)	7.7 (6.3, 9.1)	17.8 (12.6, 23)	14.6 (9.4, 19.9)	9.8 (6.6, 13)	7.6 (5.3, 9.9)	3.2 (2.1, 4.3)	0.023
	COVID-19 (1997, 2021)	4.6 (4.0, 5.3)	9.2 (6.7, 11.6)	8.3 (5.9, 10.6)	5.8 (4.3, 7.2)	4.4 (3.3, 5.5)	2.2 (1.7, 2.7)	-0.006
Nepal	Pre-COVID-19 (1996, 2016)	0.9 (0.6, 1.2)	3.0 (2.3, 3.8)	1.8 (1.2, 2.4)	1.4 (0.9, 1.9)	0.8 (0.3, 1.3)	-0.9 (-1.3, -0.5)	-0.008
	COVID-19 (1996, 2022)	0.7 (0.5, 1.0)	2.6 (2.1, 3.1)	1.6 (1.2, 2.1)	1.2 (0.7, 1.6)	0.3 (-0.2, 0.7)	-0.9 (-1.3, -0.5)	-0.041
Philippines	Pre-COVID-19 (1993, 2017)	2.0 (1.8, 2.2)	3.9 (3.4, 4.4)	2.8 (2.3, 3.2)	2 (1.6, 2.4)	1.3 (0.8, 1.7)	0.8 (0.3, 1.3)	-0.030
	COVID-19 (1993, 2022)	1.8 (1.6, 2.0)	3.3 (2.9, 3.7)	2.4 (2.0, 2.7)	1.9 (1.6, 2.2)	1.0 (0.6, 1.4)	1.2 (0.9, 1.5)	-0.025
**COVID Total**		**2.4 (2.2, 2.5)**	**3.8 (3.5, 4)**	**3.2 (3.0, 3.4)**	**2.5 (2.3, 2.7)**	**2.1 (1.9, 2.3)**	**1.3 (1.2, 1.5)**	0.021

Note: The countries included in this table collected data during the years of the COVID-19 pandemic, 2021 and 2022

Appendix B: all women of reproductive age

Table B- 1: mCPR and mDFPS reported in the first and most recent DHS round, all women

Country	All women					
	Sample		mCRP		mDFPS	
	Round *1*	Round *n*	Round *1*	Round *n*	Round *1*	Round *n*
Benin (1996, 2017-18)	5,491	15,928	3.4	11.7	8.5	28.8
Burkina Faso (1993, 2010)	6,354	17,087	4.0	14.3	9.3	39.9
Burundi (2010, 2016-17)	9,389	17,269	11.0	14.6	32.8	40.3
Cameroon (1991, 2011)	3,871	15,426	4.2	16.1	11.3	39.2
Chad (1996-97, 2014-15)	7,454	17,719	1.2	4.8	6.6	20.2
Comoros (1996, 2012)	3,050	5,329	7.8	9.9	22.7	28.8
Congo (2005, 2011-12)	7,051	10,819	13.5	22.3	22.9	38.0
Congo Dem. Rep. (2007, 2013-14)	9,995	18,827	6.7	8.1	16.1	19.5
Cote d'Ivoire (1994, 2011-12)	8,099	10,060	5.7	13.9	20.9	32.2
Ethiopia (2000, 2016)	15,367	15,683	4.7	24.9	15.3	61.4
Gambia (2013, 2019-20)	10,233	11,865	6.5	12.2	26.7	41.3
Ghana (1998, 2014)	5,691	9,396	15.3	18.2	33.8	41.2
Guinea (1999, 2018)	6,753	10,874	4.9	11.4	16.5	38.8
Kenya (1993, 2014)	7,540	31,079	20.7	39.1	41.5	70.8
Lesotho (2004, 2014)	7,095	6,621	27.6	48.5	55.2	78.9
Liberia (2007, 2019-20)	7,092	8,065	11.7	25.3	25.8	47.8
Madagascar (1997, 2008-09)	7,060	17,375	7.3	23.0	19.6	48.6
Malawi (1992, 2015-16)	4,849	24,562	6.3	45.2	16.1	73.9
Mali (1995-96, 2018)	9,704	10,519	5.0	15.4	15.4	41.4
Mozambique (1997, 2011)	8,779	13,745	5.4	12.1	18.9	36.5
Niger (1998, 2012)	7,577	11,160	4.4	11.0	19.2	40.9
Nigeria (1990, 2018)	8,778	41,821	3.8	10.5	15.2	35.7
Rwanda (1992, 2019-20)	6,551	14,634	8.6	35.1	22.8	73.7
Senegal (1997, 2019)	8,593	8,649	7.0	17.9	19.7	53.4
Sierra Leone (2008, 2019)	7,374	15,574	8.2	23.9	22.6	53.1
South Africa (1998, 2016)	11,735	8,514	49.3	47.9	79.3	79.7
Tanzania (1996, 2015-16)	8,120	13,266	11.7	27.1	31.8	55.1
Togo (1998, 2013-14)	8,569	9,480	7.9	16.7	15.0	37.4
Uganda (1995, 2016)	7,070	18,506	7.4	27.3	19.8	53.8
Zambia (1996, 2013-14)	8,021	16,411	11.2	32.5	28.9	62.6
Zimbabwe (1994, 2015)	6,128	9,955	31.1	47.9	62.8	84.8
Egypt (2005, 2014)	19,474	21,762	52.8	53.5	79.0	80.0
Morocco (1992, 2003-04)	9,256	16,798	19.7	29.0	54.6	73.4
Cambodia (2000, 2014)	15,351	17,578	11.2	26.6	33.0	56.5
Indonesia (1997, 2017)	28,810	49,627	51.2	41.4	77.1	77.0
Philippines (1993, 2017)	15,029	25,074	15.1	24.9	35.8	56.1
Timor-Leste (2009-10, 2016)	13,137	12,607	12.8	14.8	39.2	46.6
Vietnam (1997, 2002)	5,331	5,341	55.8	56.7	66.7	66.6
Bangladesh (1993-94, 2017-18)	9,640	20,127	34.2	n.a.	55.8	n.a.
India (1992-93, 2019-21)	89,777	724,105	34.2	42.7	59.6	75.0
Nepal (1996, 2016)	8,429	12,862	24.6	33.2	42.8	56.3
Pakistan (1990-91, 2017-18)	6,611	12,364	8.7	n.a.	21.3	n.a.
Bolivia (1994, 2008)	8,603	16,939	11.9	24.0	24.1	43.4
Haiti (1994-95, 2016-17)	5,356	14,371	8.9	22.3	24.5	45.4
Honduras (2005-06, 2011-12)	19,948	22,757	37.7	42.9	69.2	76.0
Nicaragua (1998, 2001)	13,634	13,060	39.0	43.9	73.9	79.0
Kyrgyz Republic (1997, 2012)	3,848	8,208	35.3	22.7	69.0	62.1
Tajikistan (2012, 2017)	9,656	10,718	17.5	19.7	51.0	52.2

Notes: n.a. = not available.

Table B- 2: mCPR and mDFPS in the richest and poorest wealth quintiles in the most recent DHS round, all women

Country	mCPR						mDFPS					
	Poorest	Richest	Poorest/ Richest				Poorest	Richest	Poorest/ Richest			
			Coeff.	p-value	Lower bound	Upper bound			Coeff.	p-value	Lower bound	Upper bound
Benin (2017-18)	0.087	0.156	0.555	0.00	0.460	0.671	0.246	0.355	0.693	0.00	0.588	0.817
Burkina Faso (2010)	0.064	0.277	0.232	0.00	0.195	0.277	0.230	0.612	0.376	0.00	0.322	0.439
Burundi (2016-17)	0.149	0.159	0.937	0.40	0.805	1.090	0.394	0.472	0.835	0.00	0.744	0.936
Cameroon (2011)	0.027	0.263	0.102	0.00	0.076	0.137	0.105	0.531	0.197	0.00	0.152	0.255
Chad (2014-15)	0.035	0.099	0.357	0.00	0.259	0.492	0.158	0.333	0.475	0.00	0.357	0.632
Comoros (2012)	0.077	0.089	0.866	0.43	0.608	1.235	0.204	0.299	0.683	0.02	0.502	0.930
Congo (2011-12)	0.101	0.296	0.340	0.00	0.287	0.402	0.184	0.500	0.368	0.00	0.316	0.429
Congo Dem. Rep. (2013-14)	0.036	0.140	0.258	0.00	0.191	0.349	0.096	0.307	0.311	0.00	0.238	0.406
Cote d'Ivoire (2011-12)	0.076	0.192	0.395	0.00	0.309	0.504	0.193	0.450	0.430	0.00	0.344	0.537
Ethiopia (2016)	0.160	0.271	0.590	0.00	0.502	0.694	0.434	0.751	0.578	0.00	0.508	0.658
Gambia (2019-20)	0.126	0.110	1.148	0.20	0.927	1.421	0.390	0.428	0.910	0.24	0.777	1.066
Ghana (2014)	0.178	0.158	1.124	0.27	0.912	1.386	0.413	0.389	1.060	0.48	0.901	1.246
Guinea (2018)	0.045	0.171	0.266	0.00	0.195	0.363	0.179	0.560	0.320	0.00	0.248	0.413
Kenya (2014)	0.235	0.407	0.578	0.00	0.530	0.630	0.474	0.788	0.601	0.00	0.550	0.657
Lesotho (2014)	0.416	0.501	0.831	0.00	0.746	0.925	0.698	0.843	0.827	0.00	0.771	0.887
Liberia (2019-20)	0.223	0.237	0.941	0.57	0.760	1.164	0.413	0.509	0.811	0.02	0.677	0.971
Madagascar (2008-09)	0.154	0.265	0.580	0.00	0.500	0.673	0.414	0.478	0.866	0.02	0.767	0.978
Malawi (2015-16)	0.422	0.423	0.999	0.98	0.940	1.062	0.700	0.765	0.915	0.00	0.881	0.951
Mali (2018)	0.100	0.203	0.492	0.00	0.380	0.636	0.263	0.551	0.478	0.00	0.383	0.597
Mozambique (2011)	0.029	0.287	0.100	0.00	0.073	0.139	0.125	0.574	0.218	0.00	0.158	0.300
Niger (2012)	0.081	0.184	0.442	0.00	0.341	0.572	0.317	0.570	0.557	0.00	0.453	0.685
Nigeria (2018)	0.035	0.169	0.206	0.00	0.171	0.247	0.187	0.441	0.424	0.00	0.365	0.492
Rwanda (2019-20)	0.408	0.266	1.532	0.00	1.414	1.661	0.754	0.704	1.070	0.01	1.015	1.128
Senegal (2019)	0.144	0.186	0.774	0.02	0.621	0.964	0.414	0.632	0.654	0.00	0.539	0.794
Sierra Leone (2019)	0.183	0.255	0.719	0.00	0.624	0.827	0.462	0.552	0.837	0.00	0.749	0.936
South Africa (2016)	0.463	0.438	1.056	0.34	0.943	1.182	0.777	0.786	0.988	0.72	0.923	1.057
Tanzania (2015-16)	0.190	0.275	0.690	0.00	0.596	0.799	0.419	0.566	0.741	0.00	0.663	0.828
Togo (2013-14)	0.136	0.200	0.681	0.00	0.560	0.830	0.307	0.458	0.672	0.00	0.564	0.799
Uganda (2016)	0.189	0.308	0.613	0.00	0.548	0.686	0.385	0.630	0.611	0.00	0.559	0.667
Zambia (2013-14)	0.248	0.336	0.739	0.00	0.664	0.822	0.488	0.762	0.641	0.00	0.592	0.693
Zimbabwe (2015)	0.487	0.453	1.073	0.08	0.992	1.162	0.794	0.895	0.887	0.00	0.849	0.927
Egypt (2014)	0.430	0.526	0.818	0.00	0.737	0.908	0.724	0.821	0.881	0.00	0.825	0.942
Morocco (2003-04)	0.296	0.281	1.053	0.25	0.964	1.150	0.722	0.719	1.003	0.90	0.948	1.062
Cambodia (2014)	0.292	0.211	1.382	0.00	1.237	1.543	0.570	0.484	1.177	0.00	1.084	1.277
Indonesia (2017)	0.421	0.350	1.203	0.00	1.143	1.265	0.784	0.708	1.106	0.00	1.075	1.139
Philippines (2017)	0.319	0.173	1.846	0.00	1.619	2.104	0.593	0.503	1.179	0.00	1.076	1.293
Timor-Leste (2016)	0.156	0.140	1.114	0.24	0.929	1.336	0.456	0.467	0.976	0.74	0.850	1.122
Vietnam (2002)	0.579	0.516	1.120	0.04	1.007	1.247	0.693	0.625	1.110	0.04	1.004	1.227
Bangladesh (2017-18)	n.a.	n.a.	n.a.	n.a.	n.a.	n.a.	n.a.	n.a.	n.a.	n.a.	n.a.	n.a.
India (2019-21)	0.388	0.434	0.893	0.00	0.880	0.907	0.697	0.759	0.919	0.00	0.910	0.929
Nepal (2016)	0.326	0.317	1.029	0.62	0.920	1.150	0.550	0.534	1.031	0.55	0.933	1.139
Pakistan (2017-18)	n.a.	n.a.	n.a.	n.a.	n.a.	n.a.	n.a.	n.a.	n.a.	n.a.	n.a.	n.a.

Country	mCPR						mDFPS					
	Poorest	Richest	Poorest/ Richest				Poorest	Richest	Poorest/ Richest			
			Coeff.	p-value	Lower bound	Upper bound			Coeff.	p-value	Lower bound	Upper bound
Bolivia (2008)	0.169	0.288	0.586	0.00	0.515	0.667	0.276	0.580	0.475	0.00	0.426	0.531
Haiti (2016-17)	0.203	0.198	1.022	0.77	0.883	1.183	0.371	0.487	0.762	0.00	0.677	0.857
Honduras (2011-12)	0.402	0.414	0.969	0.30	0.913	1.028	0.678	0.783	0.866	0.00	0.831	0.902
Nicaragua (2001)	0.370	0.428	0.864	0.00	0.793	0.942	0.633	0.836	0.757	0.00	0.718	0.799
Kyrgyz Republic (2012)	0.260	0.185	1.408	0.00	1.219	1.627	0.683	0.617	1.107	0.06	0.998	1.229
Tajikistan (2017)	0.178	0.208	0.856	0.08	0.721	1.016	0.494	0.573	0.862	0.02	0.763	0.973

Notes: Poorest/ richest ratios are not adjusted for demographic characteristics. n.a. = not available.

Table B- 3: Concentration indices (CI) for mCPR and mDFPS in the first and most recent DHS, all women

Country	mCRP		mDFPS	
	Round *1*	Round *n*	Round *1*	Round *n*
Benin (1996, 2017-18)	0.351	0.129	0.276	0.088
Burkina Faso (1993, 2010)	0.659	0.316	0.629	0.225
Burundi (2010, 2016-17)	0.118	0.000	0.103	0.026
Cameroon (1991, 2011)	0.475	0.304	0.310	0.195
Chad (1996-97, 2014-15)	0.672	0.217	0.545	0.170
Comoros (1996, 2012)	0.128	0.041	0.190	0.075
Congo (2005, 2011-12)	0.161	0.169	0.156	0.158
Congo Dem. Rep. (2007, 2013-14)	0.295	0.286	0.255	0.247
Cote d'Ivoire (1994, 2011-12)	0.381	0.190	0.287	0.181
Ethiopia (2000, 2016)	0.470	0.072	0.462	0.099
Gambia (2013, 2019-20)	0.215	-0.012	0.214	0.043
Ghana (1998, 2014)	0.119	-0.042	0.174	-0.020
Guinea (1999, 2018)	0.418	0.238	0.315	0.200
Kenya (1993, 2014)	0.227	0.063	0.208	0.072
Lesotho (2004, 2014)	0.202	0.027	0.179	0.036
Liberia (2007, 2019-20)	0.276	0.009	0.216	0.040
Madagascar (1997, 2008-09)	0.397	0.100	0.308	0.016
Malawi (1992, 2015-16)	0.347	-0.009	0.302	0.015
Mali (1995-96, 2018)	0.563	0.161	0.455	0.155
Mozambique (1997, 2011)	0.535	0.454	0.418	0.288
Niger (1998, 2012)	0.568	0.208	0.462	0.142
Nigeria (1990, 2018)	0.507	0.278	0.388	0.135
Rwanda (1992, 2019-20)	0.074	-0.075	0.126	-0.009
Senegal (1997, 2019)	0.411	0.046	0.432	0.084
Sierra Leone (2008, 2019)	0.408	0.077	0.331	0.046
South Africa (1998, 2016)	0.090	-0.008	0.071	0.007
Tanzania (1996, 2015-16)	0.319	0.056	0.250	0.049
Togo (1998, 2013-14)	0.259	0.081	0.267	0.086
Uganda (1995, 2016)	0.485	0.085	0.414	0.091
Zambia (1996, 2013-14)	0.320	0.044	0.277	0.085
Zimbabwe (1994, 2015)	0.102	-0.010	0.106	0.022
Egypt (2005, 2014)	0.083	0.041	0.030	0.026
Morocco (1992, 2003-04)	0.106	-0.021	0.112	-0.004
Cambodia (2000, 2014)	0.126	-0.072	0.138	-0.038
Indonesia (1997, 2017)	0.043	-0.043	0.013	-0.020
Philippines (1993, 2017)	0.008	-0.128	0.128	-0.032
Timor-Leste (2009-10, 2016)	0.160	-0.017	0.121	0.007
Vietnam (1997, 2002)	0.026	-0.019	0.001	-0.020
Bangladesh (1993-94, 2017-18)	n.a.	n.a.	n.a.	n.a.
India (1992-93, 2019-21)	0.155	0.022	0.082	0.016
Nepal (1996, 2016)	0.207	-0.012	0.152	-0.008
Pakistan (1990-91, 2017-18)	n.a.	n.a.	n.a.	n.a.
Bolivia (1994, 2008)	0.373	0.109	0.400	0.148
Haiti (1994-95, 2016-17)	0.212	-0.010	0.253	0.045
Honduras (2005-06, 2011-12)	0.034	0.001	0.062	0.028
Nicaragua (1998, 2001)	0.033	0.012	0.065	0.043
Kyrgyz Republic (1997, 2012)	0.033	-0.076	0.012	-0.029
Tajikistan (2012, 2017)	0.087	0.044	0.053	0.033

Notes: n.a. = not available.
